# Moonlight controls lunar-phase-dependency and regular oscillation of clock gene expressions in a lunar-synchronized spawner fish, Goldlined spinefoot

**DOI:** 10.1038/s41598-018-24538-1

**Published:** 2018-04-18

**Authors:** Yuki Takeuchi, Ryo Kabutomori, Chihiro Yamauchi, Hitomi Miyagi, Akihiro Takemura, Keiko Okano, Toshiyuki Okano

**Affiliations:** 1 Department of Electrical Engineering and Bioscience, Graduate School of Advanced Science and Engineering, Waseda University, Wakamatsu-cho 2-2, Shinjuku-ku, Tokyo, 162-8480 Japan; 2 Department of Chemistry, Biology, and Marine Science, Faculty of Science, University of the Ryukyus, 1 Senbaru, Nishihara-cho, Nakagami-gun Okinawa, 903-0213 Japan

## Abstract

Goldlined spinefoot, *Siganus guttatus*, inhabits tropical and subtropical waters and synchronizes its spawning around the first quarter moon likely using an hourglass-like lunar timer. In previous studies, we have found that clock genes (*Cryptochrome3* and *Period1*) could play the role of state variable in the diencephalon when determining the lunar phase for spawning. Here, we identified three *Cry*, two *Per*, two *Clock*, and two *Bmal* genes in *S. guttatus* and investigated their expression patterns in the diencephalon and pituitary gland. We further evaluated the effect on their expression patterns by daily interruptions of moonlight stimuli for 1 lunar cycle beginning at the new moon. It significantly modified the expression patterns in many of the examined clock(-related) genes including *Cry3* in the diencephalon and/or pituitary gland. Acute interruptions of moonlight around the waxing gibbous moon upregulated nocturnal expressions of *Cry1b* and *Cry2* in the diencephalon and pituitary gland, respectively, but did not affect expression levels of the other clock genes. These results highlighted the importance of repetitive moonlight illumination for stable or lunar-phase-specific daily expression of clock genes in the next lunar cycle that may be important for the lunar-phase-synchronized spawning on the next first quarter moon.

## Introduction

Most organisms exhibit biological rhythms by synchronizing their behavioral and physiological activities with cyclic changes in the environment. Among these rhythms of diverged period lengths, circadian rhythms are widely observed.

The molecular mechanism of the circadian clock has been well studied in contrast to those of clocks with longer (infradian rhythms) and shorter (ultradian rhythms) period lengths. The circadian clock is known to be composed of clock genes and their products^1^. In animals, positive transcriptional components CLOCK and BMAL and negative components CRYPTOCHROME (CRY) and PERIOD (PER) constitute the core negative feedback loop and drive the circadian oscillatory expression of clock genes in cooperation with sub molecular loops^2^. Multiple gene duplication events occurred in lower vertebrates such as fish species resulted in multiple paralogs of the clock(-related) genes^3^, although their functional divergence is less characterized.

In temperate zones, reproductive events of birds, fish, and insects often synchronize with seasonal changes in the environment to increase the chance of mating and decrease predation risk^4^. These seasonal breeding animals use daylengths as the principal environmental cue to decide suitable timing for reproduction^5,6^. In vertebrates, the daylength measurement (photoperiodicity) is mostly served by a circadian phase-specific photoresponse that integrates photic and circadian signals in the diencephalon^7–9^. Besides this gating mechanism of photoinduction, some animals are equipped with an internal clock called a ‘circannual clock’ that oscillates with a period of approximately one year^10–12^.

**Figure 1:**
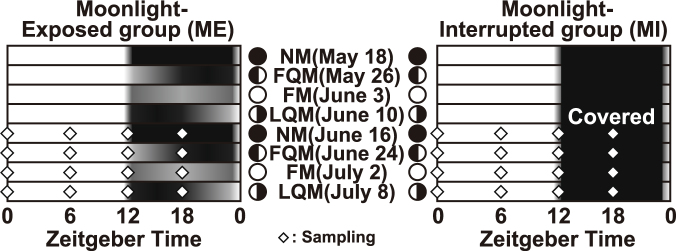
Sampling schedule and nocturnal light conditions during repetitive moonlight interruption for 2 lunar cycles. The Goldlined spinefoots were reared from May 18 to July 8 in 2015 in either the moonlight-exposed group (ME) (n = 80) or the moonlight-interrupted group (MI) (n = 64). Open diamonds indicate time points of the sample collection. Lunar phases (NM; new moon; FQM: first quarter moon; FM: full moon; LQM: last quarter moon) are indicated by schematic moon images.

Lunar-synchronized spawning has been reported in hermatypic corals, marine insects, and tropical marine fishes^13–15^. Zantke *et al*.^15^ have reported that the marine worm *Platynereis dumerilii* has an endogenous circalunar clock that controls the timing of reproduction and is possibly entrained by nocturnal light. Although the molecular mechanism of circalunar clock driving is still unknown in any species, the circalunar clock of *P. dumerilii* has been suggested to be independent of the circadian clock and to affect both the circadian behavior and the transcriptions of core clock genes^15^. On the other hand, in the Goldlined spinefoot, lunar-phase specific synchronized spawning, which occurrs at night around the first quarter moon, seemed to be strongly related to a cyclic change in moonlight illumination with a period of 29.5 days (Fig. 1); their spawning was disrupted by interrupted moonlight that occured 1 month before the expected spawning day, while the fish spawned under conditions in which they were deprived of moonlight from 2 weeks before the expected spawning day^16^. Based on these results, lunar-synchronized spawning of the spinefoot may be explainable by a mechanism controlled through an hourglass-like lunar timer, which can measure a period of less than 1 month, instead of a self-sustainable circalunar clock as seen in *P. dumerilii*.

Although the involvement of clock-related genes in the molecular mechanisms underlying lunar-related rhythmicity are far less understood, lunar phase-dependent changes in *Cryptochrome* (*Cry*) expression were observed in the Goldlined spinefoot^17^ and a lunar-synchronized spawning coral, *Acropora millepora*^18^. In Fukushiro *et al*.^17^, we reported lunar phase-dependent changes in *Cry1b* (termed as *Cry1* in ref.^17^) and *Cry3* mRNA expression in the diencephalon of the Goldlined spinefoot with an increase observed around the new moon and a decrease around the full moon. In Toda *et al*.^19^, we investigated whether the 2-week interruption in moonlight from the new moon to full moon around the spawning phase (the first quarter moon) changed the expression pattern of *Cry3*. The interruption resulted in little to no effect on the lunar phase-dependent expression of *Cry3*, indicating that *Cry3* does not just acutely respond to daily moonlight illumination but that it follows the lunar phase for at least 2 weeks. This study together with our previous results arise a question that *Cry3* and/or other circadian genes of the Goldlined spinefoot may act as signaling molecule(s) in the putative hourglass-like lunar timer which potentially contributes to spawning-lunar-phase recognition. Alternatively, it was possible that *Cry3* might constitute an unidentified lunar clock that sustainably oscillates more than two lunar phases without moonlight stimuli.

The present study aims to evaluate the above question and better elucidate the molecular mechanism underlying lunar phase-recognition of the Goldlined spinefoot. We investigated the daily and monthly patterns of *Cry*, *Per*, *Clock*, and *Bmal* expression in both the diencephalon and its downstream pituitary gland. Their expression patterns were compared with those after a 4 week moonlight interruption period. The repetitive moonlight stimuli plays a significant role in both stabilizing daily expression of circadian clock genes and triggering lunar-phase-synchronized expression of *Cry3*.

## Results

### Identification of *Cry* and *Per* paralogs in the Goldlined spinefoot

First, we conducted massive transcriptome sequencing in the brain of the Goldlined spinefoot to identify core clock gene paralogs unexamined in our previous study. Phylogenetic analyses followed by Blast search (Fig. S1A–D) revealed three unreported *Cry* paralogs (*Cry1a* [FX985477]*, Cry2a* [FX985476]*, Cry6* [FX985478]), two *Per* paralogs (*Per2a* [FX985479]*, Per3* [FX985480]), two *Clock* paralogs (*Clock1a* [FX985868], *Clock1b* [LC367223]), two *Bmal* paralogs (*Bmal1* [FX985869], *Bmal2* [LC367224]), in addition to the known paralogs *Cry1b* (AB643455), *Cry3* (AB643456), *Per1* (DQ198087), and *Per2b* (EF208027, formerly termed *Per2* in ref.^20^.

### Sampling and analysis of clock gene mRNA expression by qPCR

**Figure 2:**
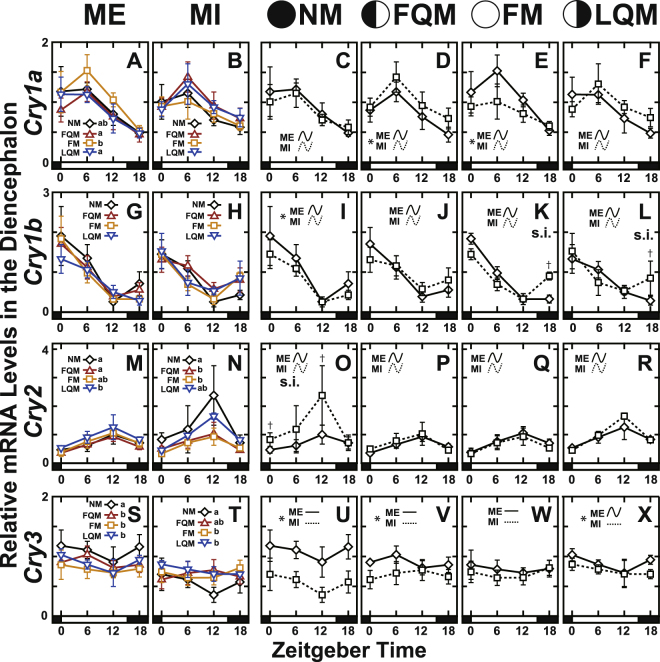
The daily expression profiles of *Cry* genes in the diencephalon of the Goldlined spinefoot after interrupted moonlight for 1 lunar cycle. The diencephalons of the fish (n = 5) were collected at ZT0, ZT6, ZT12, ZT18 on the day of the new moon (NM), first quarter moon (FQM), full moon (FM), and last quarter moon (LQM) phase (Fig. 1). The bar at the bottom of each graph represents the sunlight conditions. (Panels on the left: A,B,G,H,M,N,S and T) The daily expression profiles of genes at four lunar phases in moonlight-exposed (ME: panels A, G, M and S) and moonlight-interrupted (MI: panels B,H,N and T) groups. The different letters (a, b) in panels (A,M,N,S and T) indicate statistical differences among lunar phases (two-way factorial ANOVA followed by Tukey’s HSD test, *p* < 0.05). (Panels on the right: C–F, I–L, O–R, and U–X) The daily profiles shown on the left are replotted to compare the daily profiles of ME and MI groups at each lunar phase. Lunar phase is indicated by a schematic moon image. s.i. and asterisks indicate significant interactions and significant differences, respectively, between ME and MI analyzed using two-way factorial ANOVA followed by Tukey’s HSD test (*p* < 0.05). The statistical difference between ME and MI at each time point is indicated by a dagger (Student’s t-test or Mann–Whitney U test, *p* < 0.05). Curves indicate significant rhythmicities detected by Cosinor analysis, while lines indicate no significant rhythmicity.

**Figure 3:**
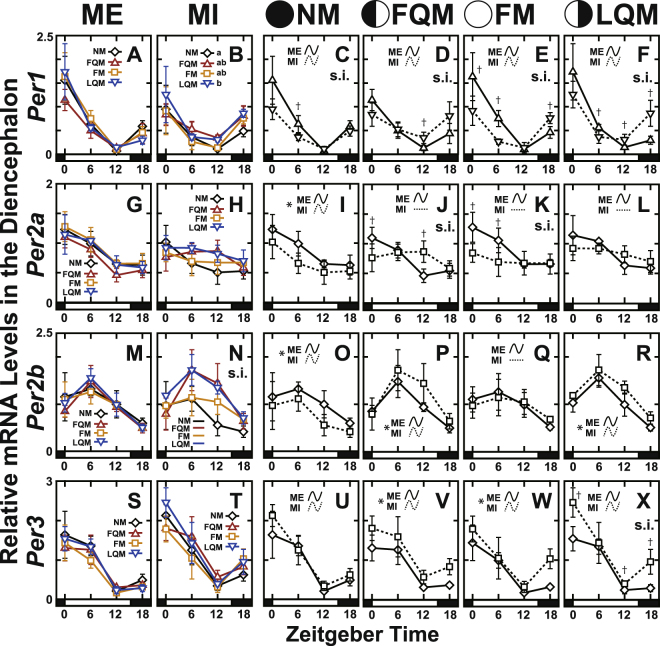
The daily expression profiles of *Per* genes in the diencephalon of the Goldlined spinefoot after interrupted moonlight for 1 lunar cycle. The diencephalons of the fish (n = 5) were collected at time points shown in Fig. 1. The bar at the bottom of each graph represents the sunlight conditions. (Panels on the left: A,B,G,H,M,N,S and T) The daily expression profiles of the genes at four lunar phases in moonlight-exposed (ME: panels A,G,M and S) and moonlight-interrupted (MI: panels B,H,N and T) groups. s.i. in panel N indicates a significant interaction. Different letters in panel B indicate statistical differences among lunar phase (two-way factorial ANOVA followed by Tukey’s HSD test, *p* < 0.05). (Panels on the right: C–F, I–L, O–R, and **U**–**X**) The daily profiles shown on the left are replotted to compare the daily profiles of ME and MI groups at each lunar phase. s.i. and asterisks indicate significant interactions and significant differences, respectively, between ME and MI groups analyzed using two-way factorial ANOVA followed by Tukey’s HSD test (*p* < 0.05). Statistical differences between ME and MI at each time point are indicated by daggers (Student’s t-test or Mann–Whitney U test, *p* < 0.05). Curves indicate significant rhythmicities detected with Cosinor analysis, while lines indicate no significant rhythmicity.

**Figure 4:**
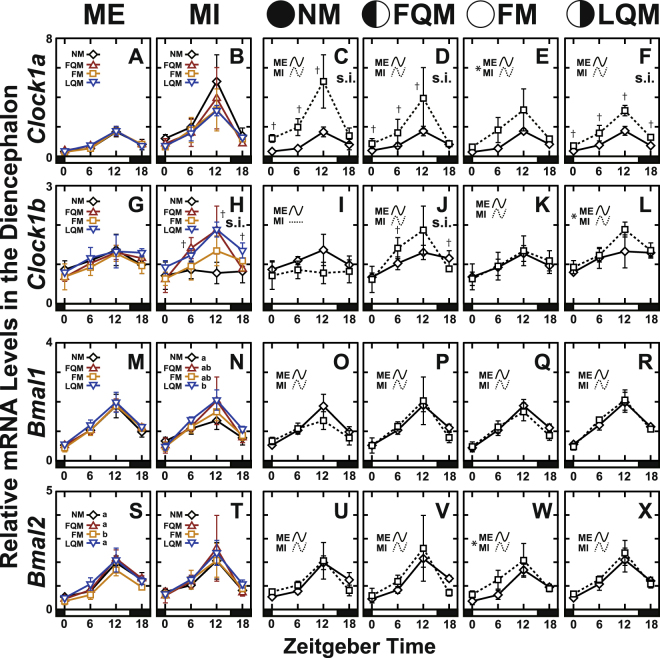
The daily expression profiles of *Clock* and *Bmal* genes in the diencephalon of the Goldlined spinefoot after interrupted moonlight for 1 lunar cycle. The diencephalons of the fish (n = 5) were collected at time points shown in Fig. 1. The bar at the bottom of each graph represents the sunlight conditions. (Panels on the left: A,B,G,H,M,N,S and T) The daily expression profiles of the genes at four lunar phases in moonlight-exposed (ME: panels A,G,M and S) and moonlight-interrupted (MI: panels B,H,N and T) groups. s.i. in panel (H) indicates a significant interaction. Different letters in panels (N and S) indicate statistical differences among lunar phase (two-way factorial ANOVA followed by Tukey’s HSD test, *p* < 0.05). (Panels on the right: C–F, I–L, O–R and U–X) The daily profiles shown on the left are replotted to compare the daily profiles of ME and MI groups at each lunar phase. s.i. and asterisks indicate significant interactions and significant differences, respectively, between ME and MI groups analyzed using two-way factorial ANOVA followed by Tukey’s HSD test (*p* < 0.05). Statistical differences between ME and MI at each time point are indicated by daggers (Student’s t-test or Mann–Whitney U test, *p* < 0.05). Curves indicate significant rhythmicities detected with Cosinor analysis, while lines indicate no significant rhythmicity.

**Figure 5:**
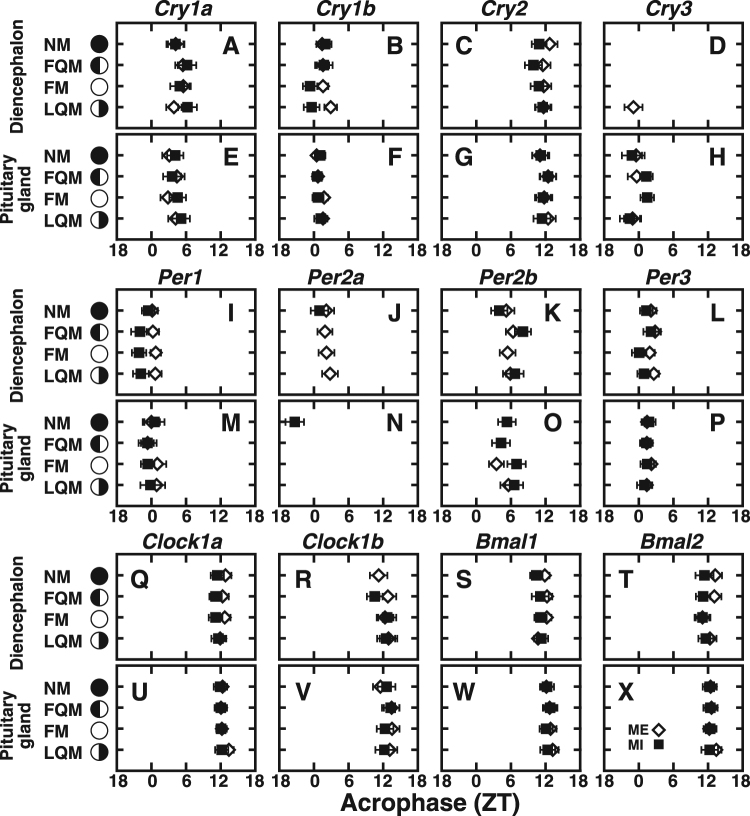
Peak phases of the clock gene expression in the Goldlined spinefoot. The acrophases of the clock gene expressions for ME and MI groups at each lunar phase (Figs 2–4, S2–S4; Tables S3, S4) estimated by Cosinor analysis are plotted. Open diamonds and filled squares show ME and MI, respectively. Error bars represent ± SEM. Schematic moon images represent lunar phase (new moon, NM; first quarter moon, FQM; full moon, FM; last quarter moon, LQM).

To investigate whether the core clock genes show lunar phase-dependent variation and whether those changes might continue in the absence of moonlight cues, rearing tanks were either repeatedly covered with a black sheet during nighttime to interrupt the moonlight (moonlight-interrupted; MI) or were exposed to natural moonlight conditions (moonlight-exposed; ME) for 2 lunar cycles (Fig. 1). We collected the diencephalon and pituitary gland at 4 representative lunar phases in the 2nd lunar cycle (Fig. 1, open diamonds) to analyze the daily expression patterns of clock genes by qPCR using specific primers (Table 1). The daily expression profiles in the diencephalon (*Cry*, Fig. 2; *Per*; Fig. 3; *Clock*, Fig. 4A–L; *Bmal*, Fig. 4M–X; statistics for all examined genes, Table S1) and the pituitary gland (*Cry*, Fig. S2; *Per*; Fig. S3; *Clock*, Fig. S4A–L; *Bmal*, Fig. S4M–X; statistics for all examined genes, Table S2) were further analyzed by the Cosinor method (Fig. 5; Tables S3 and S4).

**Table 1:** Primers used in quantitative RT-PCR analysis.

Gene name	Primer name	Sequence	Amplification efficiency[%]
*SgCry1a*	SgCry1a_qRT-PCR_F	CGCCCAGTTGAGTAATGGTC	100.86
	SgCry1a_qRT-PCR_R	GCAGAGATTCCTTGAGCGAC	
*SgCry1b* ^1^	SgCry1_RT_PCR_F	TAGAGGATTTGGACGCCAGCCTAC	97.58
	SgCry1_RT_PCR_R	CAGCCTCACTGGCTAGTTTATGGAC	
*SgCry2*	SgCry2_qRT-PCR_F	GAGGCTCTGACGAGGATAGAG	98.92
	SgCry2_qRT-PCR_R	AGCGAGTTGGCGTTCATC	
*SgCry3* ^1^	Sg Cry3 RT_PCR_F	GGTGTGGAGACTATTGTCAGAAACTCA	88.66
	Sg Cry3 RT_PCR_R	CTTCCAGCGATGGGATACTGTATAAC	
*SgPer1* ^3^	SgPer4-RTPCR-F	CCCTCCAGACAAGAGGATCTTC	100.34
	SgPer4-RTPCR-R	CCCCGCAAACTGAAAGATCT	
*SgPer2a*	SgPer2a_qRT-PCR_F1	GAACAGAAACGACCTGCTGAG	94.15
	SgPer2a_qRT-PCR_R1	TTGCCATCTGGTTTGGATG	
*SgPer2b* ^2^	SgPer2-RTPCR-F	CTGTTGGGTTACCTCCCTCA	98.65
	SgPer2-RTPCR-R	AAGCGGATCGAGGAGTGATCA	
*SgPer3*	SgPer3_qRT-PCR_F2	GATTCAGCACACACCTGAGC	101.43
	SgPer3_qRT-PCR_R2	AACCCTGTGTGTCGATCTCC	
*SgClock1a*	SgClock1a_qPCR_F2	GGTTCAGATTGCCACTAGCC	104.38
	SgClock1a_qPCR_R2	CCAGCTTGGTGAGTAGCTGAG	
*SgClock1b*	SgClock1b_qPCR_F1	CCCAGAGAGCCTTACCACAG	107.33
	SgClock1b_qPCR_R1	ACCATTCCTGGTGATGTTGG	
*SgBmal1*	SgBmal1_qPCR_F1	GTGATGACCTGATGGCAGAC	99.28
	SgBmal1_qPCR_R1	CATCAAATGAGAAGCCATCG	
*SgBmal2*	SgBmal2_qPCR_F2	CATGACCACAGTCGAACCAG	94.45
	SgBmal2_qPCR_R2	CAGACTGAATGGGCTCACAC	
*Sgβ-actin* ^1^	actin_qRT-PCR_F	CATCGCTGACAGGATGCAGAAG	97.28
	actin_qRT-PCR_R	CTCCGATCCAGACAGAGTATTTACG	
*SgEF1α* ^1^	Sg_EF1a_qRT-PCR_F	CACAGGGACTTCATCAAGAACATGATC	96.48
	Sg_EF1a_qRT-PCR_R	CGTTCTTGGAGATACCAGCCTC	
*SgPGK* ^1^	PGK_qRT-PCR_F2	CCTCAAAGTGCTCAACAACATGGAG	91.87
	PGK_qRT-PCR_R2	CTCATCGAACTTGTCAGCGGTG	

1: *SgCry1*, *SgCry3*, *Sgβ-actin*, *SgEF1α*, *SgPGK* in ref.^17^.

2: *rfPer2* in ref.^20^.

3: *rfPer* in ref.^21^.

### Daily and monthly profiles of clock gene expression under moonlight-exposed (ME) conditions

In the diencephalon, *Cry3* expression levels showed no significant daily fluctuation at most lunar phases but showed phase-dependency (Figs S2, 5D); the average daily level of *Cry3* was significantly higher at the new moon than at the other lunar phases (Fig. S2). Every examined gene except for *Cry3* showed daily variation, mostly with peaks at a fixed time of day regardless of moon phase (Fig. 5A–C,I–L,Q–T; Table S1); *Cry1a*, *Cry1b*, and all *Per* genes had peaks in the morning, while *Cry2*, *Clock1a*, *Clock1b*, *Bmal1*, and *Bmal2* had peaks in the evening. The results of *Cry1b*, *Cry3*, and *Per1* mRNA expression in ME fish were consistent with our previous observations^17,19,21^.

Importantly, in fish maintained under natural conditions, not only *Cry3* but also *Cry1a*, *Cry2*, and *Bmal2* showed lunar phase (LP)-dependent expression (p = 4.48e-06 for *Cry3*, p = 0.00317 for *Cry1a*, p = 0.00171 for *Cry2*, p = 0.00015 for *Bmal2*, LP effect in ME fish; Figs 2A,M and S4; Table S1). Expression levels of *Cry1a* and *Cry2* were higher at FM and LQM, respectively (Fig. 2A,M), than levels at other moon phases, while those of *Bmal2* were lowest at FM (Fig. S4).

In the pituitary gland, the daily profiles of clock gene expression were similar to those of the diencephalon (Figs 5, S2–S4), albeit with some differences. Cosinor analyses detected significant daily variations in every examined gene except for *Per2a* (Fig. 5N, Table S4). *Cry1a* showed significant interaction between Zeitgeber time and lunar phase (p = 0.02855, Table S2), but the profiles were similar (Fig. S2A). All of the other genes showed no lunar phase-dependent change under ME condition.

### Daily and monthly profiles of clock gene expression under moonlight-interrupted (MI) conditions

In the diencephalon of fish reared under MI conditions, most of the examined genes exhibited robust daily variation, *Cry3* and *Per2a* being the two exceptions. *Per2a* lost its rhythmicity after the FQM in MI fish (Fig. 5J). Another observation worth of noting was that the peak time of *Cry1b* was advanced according to the lunar phase progression (Fig. 5B). This may be relevant to the moonlight-interruption-dependent upregulation of *Cry1b* expression at ZT18 in FM (Fig. 2K) and LQM (Fig. 2L), implying a possible moonlight-dependent suppression.

Similarly, mRNA expression at ZT18 is upregulated by moonlight-interruption in FQM to LQM in the case of *Per1* and *Per3* (Fig. 3D–F,V–X), causing phase advances of peak time (Fig. 5I,L) as observed in *Cry1b* (Fig. 5B). *Cry1a* expression levels were significantly lower in MI fish than ME fish during the full moon phase (Fig. 2E), suggesting that nocturnal moonlight may upregulate the expression level of *Cry1a* during the daytime.

**Figure 6:**
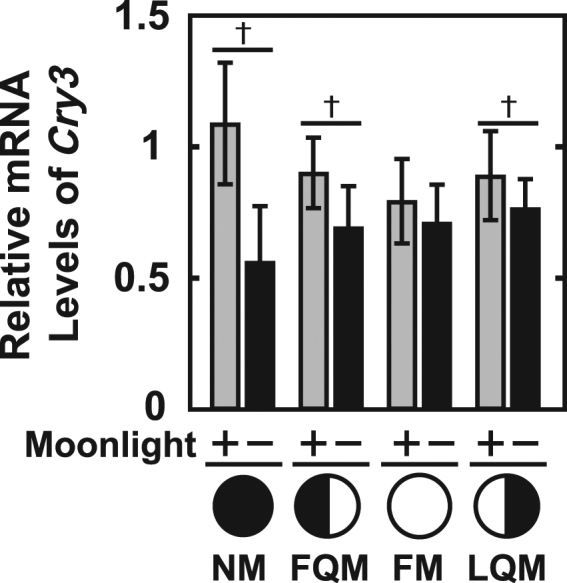
Effects of repetitive moonlight interruption on the lunar phase-dependency of *Cry3* gene expression in the diencephalon of the Goldlined spinefoot. The lunar phase-dependent changes in *Cry3* expression in moonlight-exposed (+) and moonlight-interrupted (−) groups. The relative expression levels of *Cry3* in ME and MI (Fig. 2M,N) groups were respectively averaged at each lunar phase. Because significant interactions between nocturnal light conditions (ME and MI) and lunar phase (NM, FQM, FM, and LQM) were detected with two-way factorial ANOVA (*p* < 0.05), the statistical differences between ME (n = 20) and MI (n = 16) groups at each lunar phase were analyzed using the Student’s t-test as indicated by daggers (*p* < 0.05).

*Cry1b*, *Cry2*, *Cry3*, *Per1*, *Per2a*, *Per2b*, and *Clock1a* showed moonlight-interruption-dependent changes in the daily expression profile even in the absence of moonlight at NM (Figs 2I,O,U, 3C,I,O and 4C). This indicates that daily rhythms of many clock(-related) genes are affected by moonlight in the preceding cycle (Fig. 1, May 18-Jun 10). MI fish had significantly lower *Cry3* expression levels at NM, FQM, and LQM (Fig. 2U,V,X, Fig. 6) and higher *Clock1a* expression levels at all four lunar phases (Fig. 4C–F).

Some genes (*Per1* (Fig. 3B) *Per2b* (Fig. 3N), *Clock1b* (Fig. 4H), and *Bmal1* (Fig. 4N)) showed moon phase-dependency in the expression levels under MI condition but not under ME condition, suggesting that repetitive moonlight-interruption for 1 lunar cycle may affect on the regular daily oscillation of circadian clock in the diencephalon of Goldlined spinefoots.

In the pituitary gland, *Cry2*, *Per2a*, and *Per2b* showed lunar-phase-dependent changes (Figs S2N, S3H and S3N), but the interruption of moonlight seemed to have little effect on clock gene peak expressions. *Cry1a*, *Per2a*, and *Per2b* levels were significantly higher in MI fish than ME fish (Figs S2C–F, S3I–L,O–R), suggesting that moonlight may have inhibitory effects on their expression. In contrast to the diencephalon, no lunar phase-dependent change in *Cry3* expression was observed in the pituitary gland under ME or MI conditions (Fig. S2S,T).

### Acute effects of moonlight illumination around waxing gibbous moon on clock gene mRNA expressions

As mentioned above, daily changes in clock gene expressions were observed in the diencephalon and pituitary gland, some of which may be modulated by moonlight. In particular, the nocturnal mRNA expression (ZT18) of *Cry1b* (Figs 2K,L, S2K), *Per1* (Figs 3D–F, S3E), and *Per3* (Figs 3W,X, S3W) were likely increased by moonlight interruption in both the diencephalon and pituitary gland. These modulations were observed during the FM and LQM but not the NM or FQM when the intensity of moonlight illumination were much lower than the FM.

**Figure 7:**
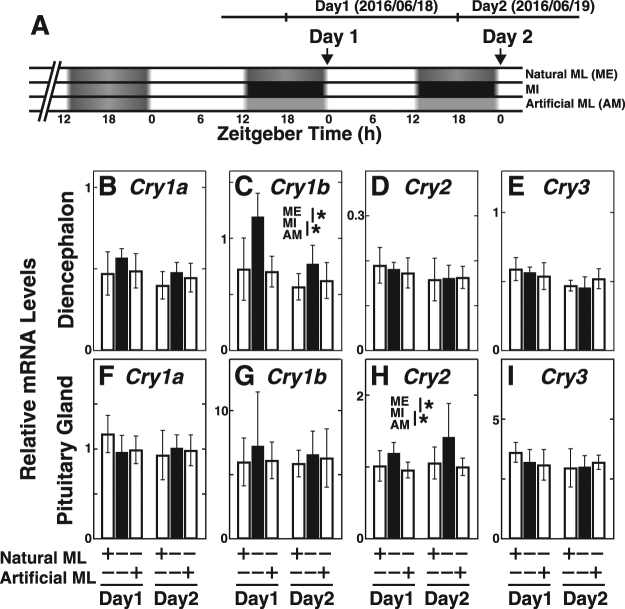
Acute effects of interrupted moonlight on *Cry* gene expressions in the diencephalon and the pituitary gland. (**A**) Sampling schedule and nocturnal light conditions during the moonlight interruption experiment around the waxing gibbous moon. Before the onset of the experiment, the Goldlined spinefoot fish were divided into three groups: moonlight exposed (ME, n = 12), moonlight interrupted (MI, n = 11), and artificial moonlight exposed (AM, n = 12) groups. The fish in each group were reared for 2 days around the waxing gibbous moon in June 2016 (full moon was June 20). The fish were randomly taken from each group at ZT0 on Day1 and 2 as indicated by arrows, and the diencephalons and pituitary glands were collected (each n = 5 except for MI on Day2 [n = 4]). (**B**–**I**) The relative expression levels of *Cry1a* (**B**,**F**), *Cry1b* (**C**,**G**), *Cry2* (**D**,**H**), and *Cry3* (**E**,**I**) in the diencephalon (**B**–**E**) and pituitary gland (**F**–**I**) under three different nocturnal conditions. Presence or absence of natural moonlight and artificial moonlight is indicated by + or − along the x-axis. Asterisks indicate significant differences among nocturnal light conditions (two-way factorial ANOVA followed by Tukey’s HSD test, *p* < 0.05).

To clarify whether such moonlight interruption-dependent elevation was an acute response to the absence of moonlight or a slow response to the repetitive interruptions of moonlight illumination, the effect of short term moonlight deprivation (1 or 2 nights) on the clock gene expression was evaluated around the waxing gibbous moon, when the intensity of moonlight illumination dramatically increases (Figs 7 and S5). Interestingly, expression levels of *Cry1b* (Fig. 7C) and *Cry2* (Fig. 7H) were significantly higher in moonlight-interrupted fish than in natural moonlight-exposed or artificial moonlight-exposed fish. *Per* expressions in the diencephalon (Fig. S5A–D) showed a similar trend, albeit only on Day 1. On the other hand, neither *Cry3*, *Clock* nor *Bmal* genes in the diencephalon (Figs 7E, S5I–L) nor any of the examined genes in the pituitary gland (Fig. S5E–H,M–P) showed such moonlight interruption-dependent elevation.

## Discussion

Our previous studies implied that *Cry3* acts as a possible state variable internally expressing lunar-phase in an hourglass-like timer mechanism or a self-oscillating lunar clock underlying the lunar phase-specific spawning of the Goldlined spinefoot^17,19^. In the present study, we aimed to answer this question through comparative measurements of the daily expression profiles of *Cry, Per, Clock*, and *Bmal* genes at the four principle lunar phases in fish maintained under natural conditions or in the absence of moonlight illumination for over 1 lunar cycle.

The lunar phase dependency of *Cry3* expression was markedly changed after repetitive deprivation of moonlight for over 1 month (Fig. 6), which contrasts with the 2-week interruption from the NM to FQM that had no such effect^19^. *Cry3* is less likely to be a circalunar clock element, in which case it would exhibit lunar phase-dependent oscillatory expression even in the absence of external cues. The present results suggest that the lunar phase-dependent change in *Cry3* expression could be regulated by moonlight signals from the previous lunar cycle, possibly preceding spawning. Thus, *Cry3* could constitute an hourglass-like timer with a period of one lunar cycle, although its association with lunar-response is yet unclear.

According to the phylogenetic analysis of vertebrate CRY, CRY3 of both zebrafish and the Goldlined spinefoot were classified into the vertebrate CRY2 cluster (Fig. S1A) which is suggested to operate in the circadian clock. Ishikawa *et al*.^22^ has reported that the inhibition of CLOCK:BMAL1-mediated transcriptional activity by zebrafish CRY3 (zCRY3) was weaker than that of zCRY1a and zCRY2b, both of which are classified into the vertebrate CRY1 cluster. This, together with the present observation, led us to speculate that CRY3 may retain a non-circadian function in addition to the established circadian function. In particular, SgCRY3 may play a role as a modifier of circadian clock inhibiting the circadian core elements according to the moon phase in the Goldlined spinefoot.

Previous study performed by Takemura *et al*.^16^ suggested that lunar-synchronized spawning of the Goldlined spinefoot requires moonlight signals from the previous lunar cycle before spawning. The synthesis and secretion of a maturation-inducing steroid hormone, 17α, 20β-dihydroxy-4-pregnen-3-one (DHP) is constitutively activated from the new moon to the first quarter moon during reproductive season^23^. Thus, moonlight signals are considered to play a key role in triggering sexual maturation for spawning in the next lunar cycle, and hence an hourglass-like timer may be reset by moonlight signals to contribute to the recognition of the timing of spawning within one lunar cycle. The expression manner of *Cry3* that changed upon the interruption of moonlight illuminations in the last cycle (Fig. 6) seems to fit well with these physiological findings where we speculate that *Cry3* is involved in the hourglass-like lunar timer.

The comparative investigations of clock gene expressions in the presence (ME) and absence (MI) of moonlight stimuli unexpectedly highlighted important observations. Many clock genes showed lunar phase-dependent daily profiles under MI condition, while rather stable profiles were shown under ME condition (Figs 2N,T, 3B,N, 4H, S2N, S3H,N). We believe that these differences are not artifacts of covering the tanks with sheets, because the difference between ME and MI is whether the fish were repeatedly covered by clear or black sheets, respectively. Although the involvement of clock genes in the lunar timer nor circalunar clock is yet to be elucidated, these results suggest significant association between the clock gene expression and moonlight which periodically changes both in intensity and duration (timing of moonrise and moonset) in accordance with the moon phase (Fig. 1). The moonlight stimuli may be necessary for stable daily expression of clock genes over the lunar cycle in the Goldlined spinefoot.

The light responsiveness of clock gene expression was additionally examined by investigation of the acute effect of moonlight interruption on clock gene expression around the waxing gibbous moon (Fig. 7). *Cry1b* and *Cry2* were likely induced by interrupted moonlight (Fig. 7C,H), and the possible light-dependent repression was mimicked by illumination from artificial moonlight in a diencephalon-specific manner. It is interesting to assume that the moonlight-dependent downregulation of *Cry1b* and *Cry2* may contribute to the upregulation of *Cry1a* (Fig. 2E) at the FM and *Cry3* at the following NM (Figs 2U, 6) in the diencephalon. Further studies including promoter analyses of these clock genes and genome-wide transcriptome analyses would assist in elucidation of the complex molecular networks underlying the lunar-phase dependent change in *Cry3* expression.

Because the luminosity of moonlight (less than 1 lux) is a lot lower than that of sunlight, moonlight cues are probably detected by a highly light-sensitive organ such as the eyes and/or the pineal gland in the Goldlined spinefoot. Melatonin is synthesized mainly in these organs, the production of which is typically accelerated at night^24^. It is widely known that melatonin production is inhibited by external light signals in vertebrates, including fish^25,26^. In the Goldlined spinefoot, this nocturnal elevation of melatonin production in the eyes and the pineal gland was reported to be suppressed by moonlight^16^. This result implies that lunar phase information can be encoded into lunar changes in the daily plasma melatonin levels that in turn can be detected by *Cry* expressing cells that express melatonin receptors in the diencephalon and pituitary gland. In this regard, the expression of a melatonin receptor gene, MT1, was found in the mediobasal region of the diencephalon of the European seabass^27^. This likely overlaps with the location where we previously reported immunopositive reactions to anti-CRY3 antibody in the Goldlined spinefoot^19^. In addition, it is interesting to note that the CRY3 immunopositive cells in the Goldlined spinefoot diencephalon resemble CSF-contacting neurons called “deep brain photoreceptors”. In the pigeon and toad, the deep brain photoreceptors are shown to express opsin proteins that may detect external sunlight directly and operate as a sensor for detecting seasonal change in photoperiod^28–30^. In the case of the Goldlined spinefoot, a similar mechanism may exist in CRY3-expressing cells of the diencephalon that are directly sensitive to sunlight for the photoentrainment of clock genes. Such a mechanism could discriminate and integrate signals of sunlight and moonlight in the cell, which could help to determine the timing of spawning.

The present study along with our previous studies provide an insight that *Cry3* of the Goldlined spinefoot could be an internal signal for recognizing external lunar phase. *Cry3* might be involved in forming a putative hourglass-type lunar timer that regulates spawning associated with the lunar cycle in the Goldlined spinefoot. However, we cannot rule out the possibility that the moonlight-dependent changes in clock gene expression in the diencephalon might be due to systemic changes such as oocyte development or sexual maturation that is initiated by FM light. Also, it is still possible that the Goldlined spinefoot might have a self-sustainable circalunar clock that is rather independent of circadian clock as shown in *P. dumerilii*.

The next step is to examine the impact of moonlight on nocturnal levels of melatonin and clock gene expression in the diencephalon as well as to analyze the moonlight-dependent transcription regulation on the promoter of clock genes such as *Cry1a* and *Cry3*. Physiological and molecular biological approaches would also be desired such as the diencephalon-specific induction and knockdown of *Cry3* and *ex vivo* culturing of the diencephalon to test responses to melatonin and light.

## Materials and Methods

### Experimental animals

Juvenile Goldlined spinefoots were originally collected from the Teima river (26°33′25.8′N 128°04′12.3′E) and the Manna river (26°40′41.4′N 127°53′15.9′E), which are located in the northern part of Okinawa island, Japan. They were reared under a natural photoperiod in concrete tanks (10 metric tons capacity) with running seawater at Sesoko Station, Tropical Biosphere Research Center, University of the Ryukyus, and were fed daily with commercial pellets (EP1, Marubeni Nisshin, Tokyo, Japan). Fish aged 3–5 years, with a body mass ranging from 162–792 g, were used in the present study. All experiments were conducted in accordance with the guidelines of WASEDA University. All protocols were approved by the Committee for the Management of Biological Experiment at WASEDA University, and experimental animal care was conducted under permission from the Committee for Animal Experimentation at the School of Science and Engineering at Waseda University (permission # WD15–060; WD16-056). Animal experiments were conducted under permission from Sesoko Station Tropical Biosphere Research Center at the University of the Ryukyus (permission # 20150518-0708; 20160531-0619). The fish were anesthetized deeply with 2-phenoxyethanol (Kanto Chemical, Tokyo, Japan) and euthanized by decapitation.

### Massive sequencing of transcriptome in the brain of the Goldlined spinefoot

Total RNA was extracted from the telencephalon, diencephalon, and optic tectum of 3–5 year old Goldlined spinefoots reared under natural conditions. Total RNA was collected at ZT0, ZT6, ZT12, and ZT18 (n = 1 each) and pooled. Samples were sent to Eurofin Genomics (Tokyo, Japan) for cDNA library construction and transcriptome sequencing using Illumina Hiseq. 2500 (read length 2 × 125 bp). After trimming the bases from the 5′end and 3′end of each read with low quality (Q <25) adapter sequences using Trimmomatic (ver. 0.3.6, ref.^31^), trimmed reads shorter than 90 bases and of low averaged quality (Q <25) were removed by PRINSEQ lite (ver. 0.20.4, ref.^32^). The cleaned raw reads were then assembled with Trinity software (ver. 2.3.2, ref.^33^) using default parameters. To search *Cry* and *Per* paralogs of the Goldlined spinefoot against the assembled sequences, the tBlastn program^34^ was utilized (E-value < 0.01) on zebrafish CRY and PER protein sequences as queries.

### Repetitive interruptions in moonlight illumination for 2 lunar cycles

Moonlight irradiation was interrupted for 2 lunar cycles (from the new moon on May 18, 2015 to the last quarter moon on July 8, 2015) at Sesoko Station (Fig. 1). The goldlined spinefoots (n = 144) were divided into two groups (the moonlight-interrupted group, MI, and moonlight-exposed group, ME) and reared in outdoor tanks (1 metric ton capacity) equipped with running seawater and an aeration system. The tank holding the MI group was covered with a black plastic sheet during the night hours (19:20-5:40) to prevent moonlight penetration. The tank holding the ME group was covered with a clear plastic sheet that allowed moonlight to filter in at night. After rearing the fish under these conditions for 1 lunar cycle, samples were taken from the diencephalon and pituitary gland of MI (n = 4) and ME (n = 5) fish at ZT0 (6:00), ZT6 (12:00), ZT12 (18:00), and ZT18 (24:00) on the day of the new moon (June 16), first quarter moon (June 24), full moon (July 2), and last quarter moon (July 8). Collected samples were immersed in RNAlater (Thermo Fisher Scientific, MA, USA) at 4 °C for at least 24 hours, and then stored at −80 °C until total RNA extraction. Samplings at ZT0, ZT6, and ZT12 were conducted under fluorescent lighting (Toshiba, FHF32EX-N-H 32 W, approximately 800 lux) and sampling at ZT18 was done under dim red LED lighting (2.5 W red LED; OPTILED, Optiled Lighting International Ltd., Kwun Tong, Hong Kong, λ_peak_ = 627 nm).

### Two-night interruptions of moonlight illumination

Fish aged three to five years old (n = 35) were divided into three groups (Moonlight exposed group, ME, n = 12; Moonlight interrupted group, MI, n = 11; and the Artificial moonlight-irradiated group, AM, n = 12) and kept in outdoor 200 L polyethylene tanks with running seawater and aeration under natural conditions before the onset of the experiment (May 31, 2016). After acclimation in the tanks for 17 days, the MI and AM groups were covered with a black plastic sheet to prevent moonlight illumination at night (ZT12-18) for 2 days beginning from the day of the waxing gibbous moon (moon age = 12)(Fig. 7A). AM fish were exposed to illumination from hand-made LED lights designed to mimic moon light (1 lux on the water surface; Fig. S6) while the tank was covered with a black sheet at night. The ME tank was covered with a clear plastic sheet to allow moonlight to shine into the tank as a control. Fish (n = 4–5) from each group were taken from the tanks randomly on Day1 at ZT0 (n = 5), and on Day2 at ZT0 (n = 5 except for MI [n = 4]), and the diencephalon and pituitary gland of each fish were collected. The samples were stored as mentioned above until total RNA extraction. Throughout the experimental period (the time of sunrise was 05:40 JST and that of sunset was 19:24 JST), the weather was conducive to having enough moonlight at night.

### Quantitative RT-PCR

Total RNA was extracted from the collected samples using Trizol (Thermo Scientific) according to the manufacturer’s instructions. The quantity of total RNA was assayed spectrophotometrically at 260 and 280 nm. ReverTra Ace qPCR Master Mix with gDNA Remover (TOYOBO) was used to digest genomic DNA contaminating the total RNA and to synthesize cDNA from 1 μg of total RNA according to the manufacturer’s instructions. Quantitative RT-PCR analyses were performed using StepOnePlus (Applied Biosystems) along with a Fast SYBR Green Master Mix (Applied Biosystems). The primers for quantitative RT-PCR are shown in Table 1. The PCR products were subjected to 3% agarose gel electrophoresis and analyzed using a Typhoon 9410 scanner (GE healthcare). The relative mRNA expression levels of target genes were calculated using the ΔΔCt method, and the reference gene was virtually defined as the average of the threshold cycles (Ct) for *SgPGK* (AB643458), *SgEF1*α (AB643459) and *Sgβ-actin* (AB643460) as described in ref.^19^.

### Statistics

In moonlight interruption experiments conducted in 2015 (Figs 2–4 and S2–S4), a three-way interaction involving the effect of lunar phase (NM, FQM, FM, LQM), Zeitgeber time (ZT: 0, 6, 12, 18), and nocturnal light condition (NLC; exposed, interrupted) was analyzed using three-way ANOVA. Because there was no significant interaction detected in three-way ANOVA analysis, a simple interaction between LP and ZT in each NLC and between NLC and ZT in each LP was analyzed using two-way factorial ANOVA (Tables S1 and S2). When a significant simple interaction was detected, a simple-simple main effect was analyzed using the Student’s t-test or Mann–Whitney U test according to results of the F test to compare the expression levels at each time point. The rhythmicity and acrophase of the daily expression profiles were analyzed using the Cosinor method on CircWave ver. 1.4 [ref.^35^] at a fixed period of 24 h. The acute effects of nocturnal light treatment (Fig. 7) were evaluated by two-way factorial ANOVA. Values of *p* < 0.05 were considered statistically significance for all analyses. Error bars represent ± SD (Figs 2–4, S2–S4).

## Electronic supplementary material

Supplementary Information
