# Supplementary material for: Moonlight controls lunar-phase-dependency and regular oscillation of clock gene expressions in a lunar-synchronized spawner fish, Goldlined spinefoot
Source: Sci Rep. 2018 Apr 18;8:6208. doi: 10.1038/s41598-018-24538-1 (PMC5906463; doi:10.1038/s41598-018-24538-1)
Supplement: Supplementary file 1 — Supplementary Information [file 41598_2018_24538_MOESM1_ESM.pdf]

Supplementary information

Moonlight controls lunar-phase-dependency and regular oscillation of clock gene expressions in a lunar-synchronized spawner fish, Goldlined spinefoot

Yuki Takeuchi[1], Ryo Kabutomori[1], Chihiro Yamauchi[2], Hitomi Miyagi[2], Akihiro Takemura[2], Keiko Okano[1], Toshiyuki Okano[1]*

Author affiliations:
[1] Department of Electrical Engineering and Bioscience, Graduate School of Advanced Science and Engineering, Waseda University, Wakamatsu-cho 2-2, Shinjuku-ku, Tokyo 162-8480, Japan

[2] Department of Chemistry, Biology, and Marine Science, Faculty of Science, University of the Ryukyus, 1 Senbaru, Nishihara-cho, Nakagami-gun, Okinawa 903-0213, Japan

*Corresponding author:
Dr. Toshiyuki Okano
Department of Electrical Engineering and Bioscience, Graduate School of Advanced Science and Engineering, Waseda University, Wakamatsu-cho 2-2, Shinjuku-ku, Tokyo 162-8480, Japan
Tel and Fax: +81-3-5369-7316
E-mail: okano@waseda.jp

Running title: Effect of moonlight on clock gene expression in lunar-responsive fish

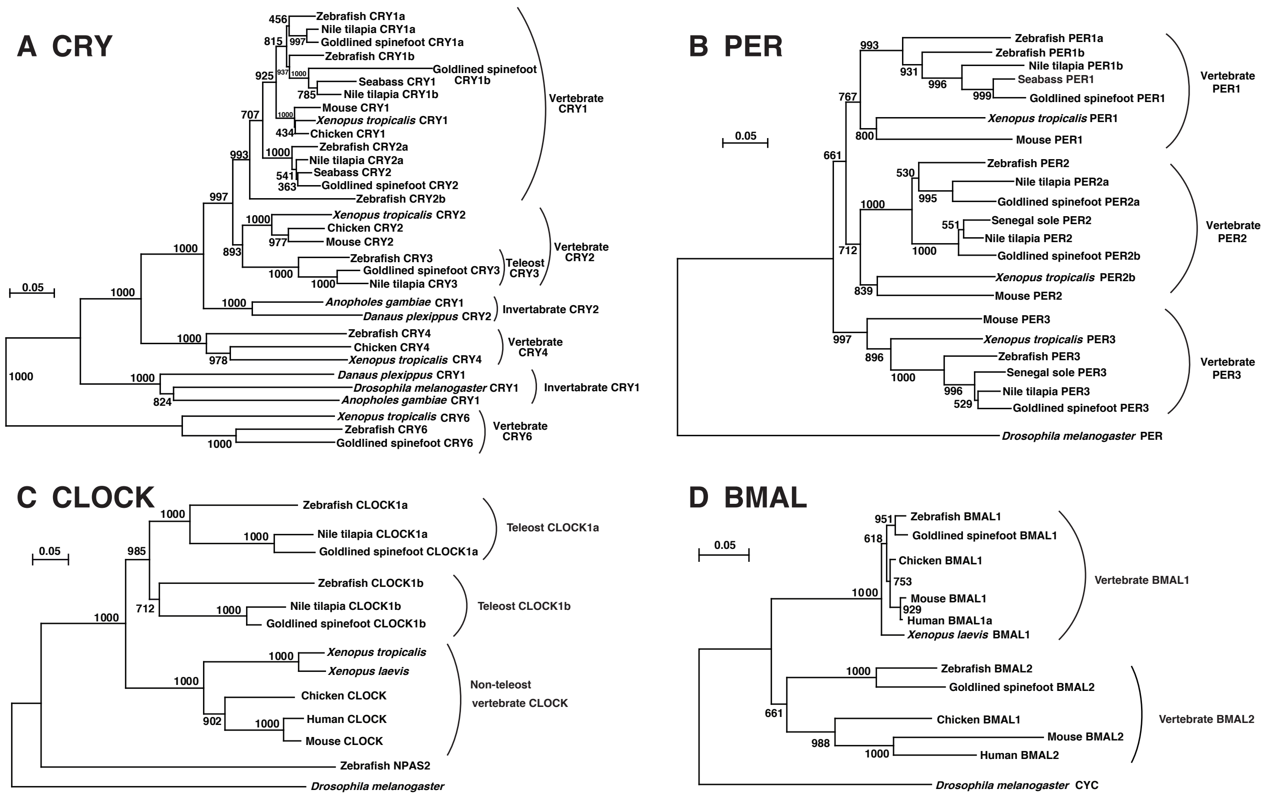

Figure S1. Phylogenetic trees of CRY (A), PER (B), CLOCK (C), and BMAL (D) family proteins. The amino acid sequences of the Goldlined spinefoot CRY, PER, CLOCK, and BMAL candidates were deduced using the ORF Finder program (NCBI, http:// www.ncbi.nlm.nih.gov/). Multiple alignments with the other clock proteins (accession nos. of are shown in Tables S5-S8) and the construction of a phylogenetic tree using the Neighbor-Joining method (Saitou and Nei, 1987) were conducted using CLUSTAL W (http://clustalw.ddbj.nig.ac.jp/top-e.html). One thousand bootstrap repetitions were performed, and values are shown at the inner nodes. The scale bar is calibrated in substitutions per site.

Reference

Saitou N, Nei M (1987) The neighbor-joining method: a new method for reconstructing phylogenetic trees. Mo.l Biol. Evol. 4: 406–425

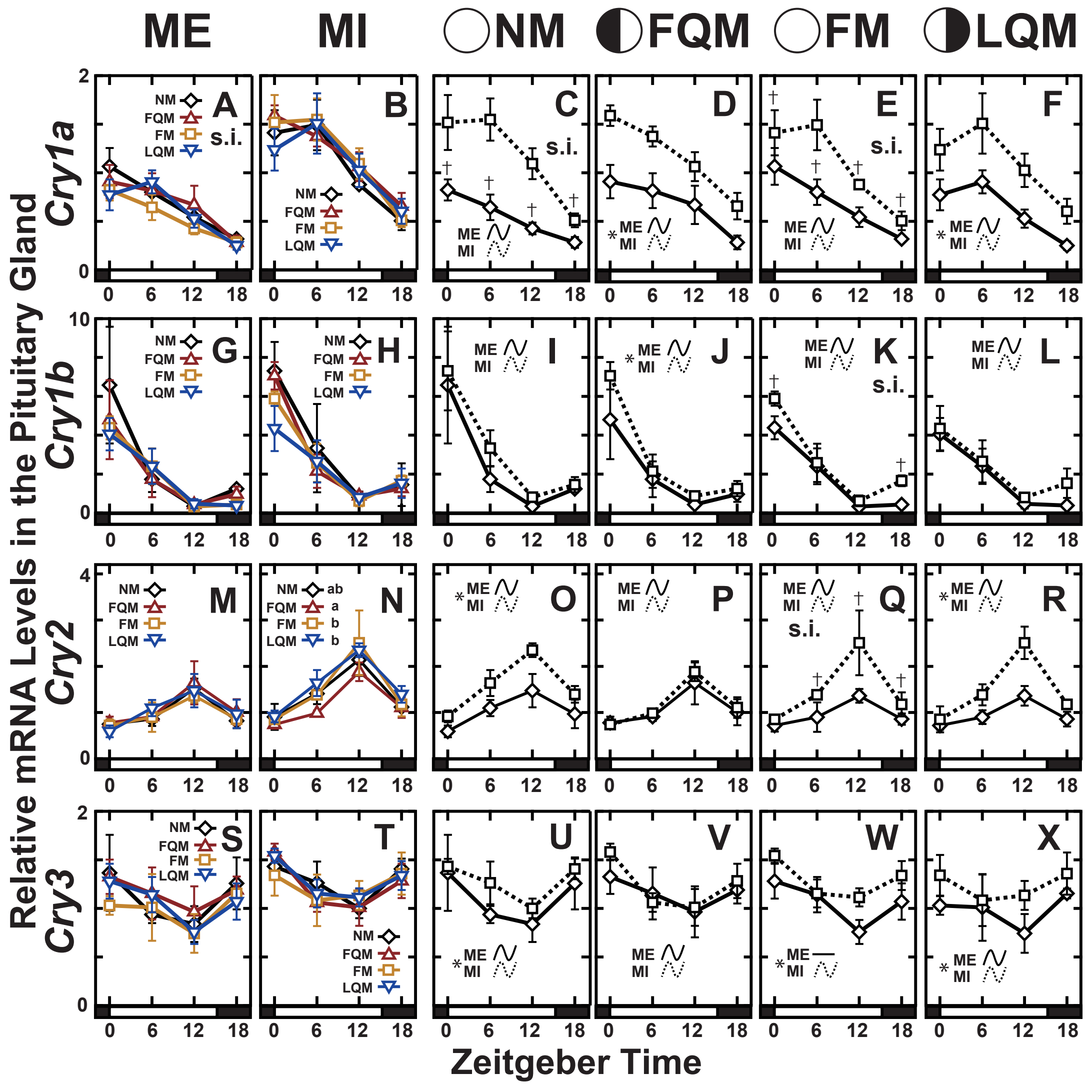

Figure S2. The daily expression profiles of *Cry* genes in the pituitary gland of the Goldlined spinefoot after interrupted moonlight for 1 lunar cycle. The pituitary glands of the fish (n = 5 except for NM-ZT18 and LQM-ZT6 in the ME group, and n=4 in the MI group) were collected at the time points shown in Fig. 1. The bar at the bottom of each graph represents the sunlight conditions. (Panels on the left: A, B, G, H, M, N, S, and T) The daily expression profiles of the genes at four lunar phases in moonlight exposed (ME) and moonlight interrupted (MI) groups. s.i. in panel A indicates a significant interaction. Different letters in panels N indicate statistical differences among lunar phase (two-way factorial ANOVA followed by Tukey's HSD test, $p < 0.05$). (Panels on the right: C-F, I-L, O-R, and U-X) The daily profiles shown on the left are replotted to compare the daily profiles of ME and MI groups at each lunar phase. Lunar phases are indicated by schematic moon images. s.i. and asterisks indicate significant interactions and significant differences, respectively, between ME and MI analyzed using two-way factorial ANOVA followed by Tukey's HSD test ($p < 0.05$). Statistical differences between ME and MI at each time point are indicated by daggers (Student's t-test, $p < 0.05$). Curves indicate significant rhythmicities detected with Cosinor analysis, while lines indicate no significant rhythmicity.

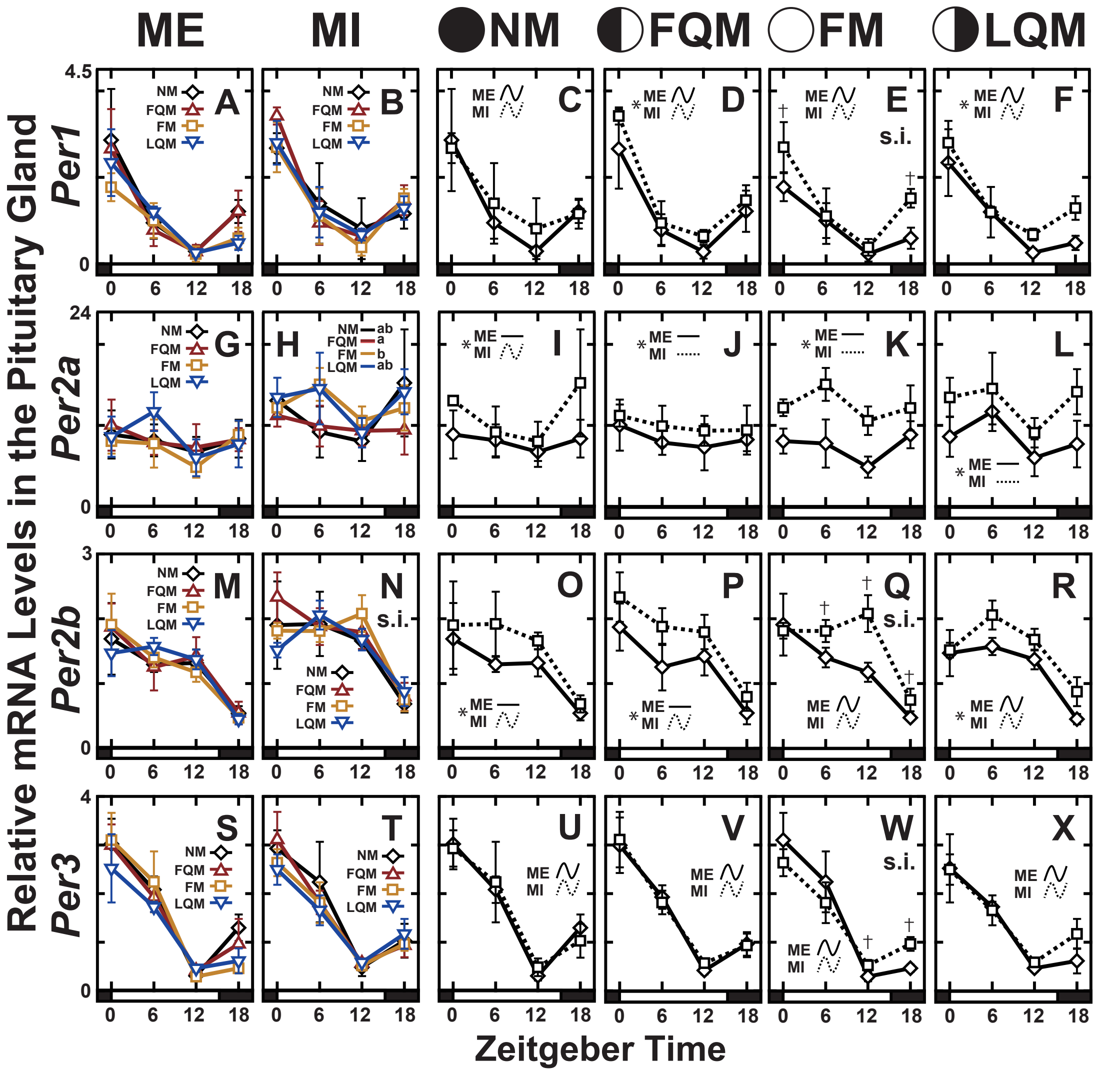

Figure S3. The daily expression profiles of *Per* genes in the pituitary gland of the Goldlined spinefoot after interrupted moonlight for 1 lunar cycle. The pituitary glands of the fish (n = 5 except for NM-ZT18 and LQM-ZT6 in ME group, and MI group [n = 4]) were collected at the time points shown in Fig. 1. The bar at the bottom of each graph represents the sunlight conditions. (Panels on the left: A, B, G, H, M, N, S, and T) The daily expression profiles of the genes at four lunar phases in moonlight-exposed (ME: panels A, G, M, and S) and moonlight-interrupted (MI: panels B, H, N, and T) groups. s.i. in panel N indicates significant interaction. The different letters in panel H indicate statistical differences among lunar phases (two-way factorial ANOVA followed by Tukey's HSD test, $p < 0.05$). (Panels on the right: C-F, I-L, O-R, and U-X) The daily profiles shown on the left are replotted to compare the daily profiles of ME and MI groups at each lunar phase. s.i. and asterisks indicate significant interactions and significant differences, respectively, between ME and MI groups analyzed using two-way factorial ANOVA followed by Tukey's HSD test ($p < 0.05$). Statistical differences between ME and MI groups at each time point are indicated by daggers (Student's t-test or Mann–Whitney U test, $p < 0.05$). Curves indicate significant rhythmicities detected with Cosinor analysis, while lines indicate no significant rhythmicity.

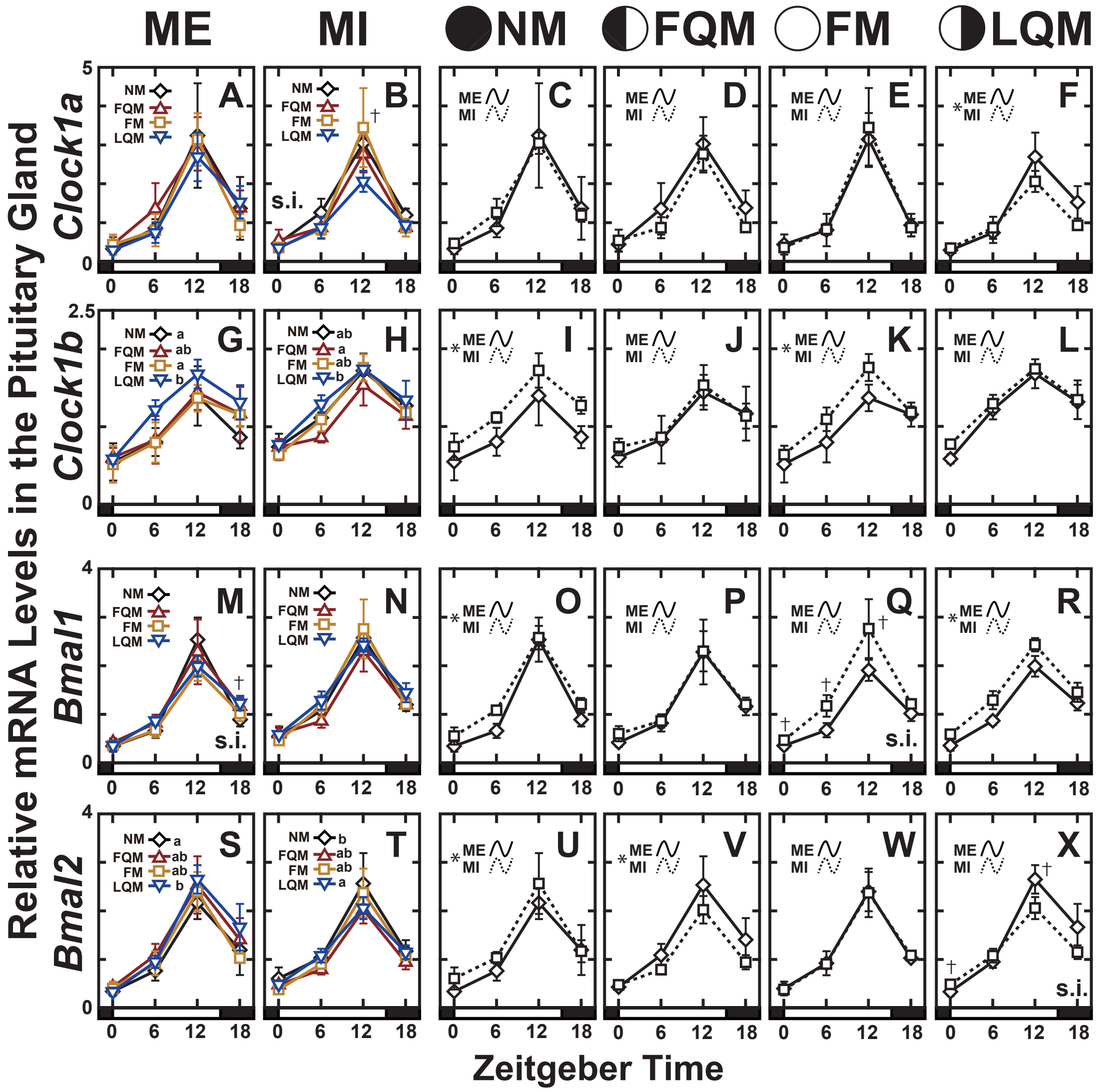

Figure S4. The daily expression profiles of *Clock* and *Bmal* genes in the pituitary gland of the Goldlined spinefoot after interrupted moonlight for 1 lunar cycle. The pituitary glands of the fish (n = 5 except for NM-ZT18 and LQM-ZT6 in ME group, and MI group [n = 4]) were collected at the time points shown in Fig. 1. The bar at the bottom of each graph represents the sunlight conditions. (Panels on the left: A, B, G, H, M, N, S, and T) The daily expression profiles of the genes at four lunar phases in moonlight-exposed (ME: panels A, G, M, and S) and moonlight-interrupted (MI: panels B, H, N, and T) groups. (Panels on the right: C-F, I-L, O-R, and U-X) The daily profiles shown on the left are replotted to compare the daily profiles of ME and MI groups at each lunar phase. Curves indicate significant rhythmicities detected with Cosinor analysis.

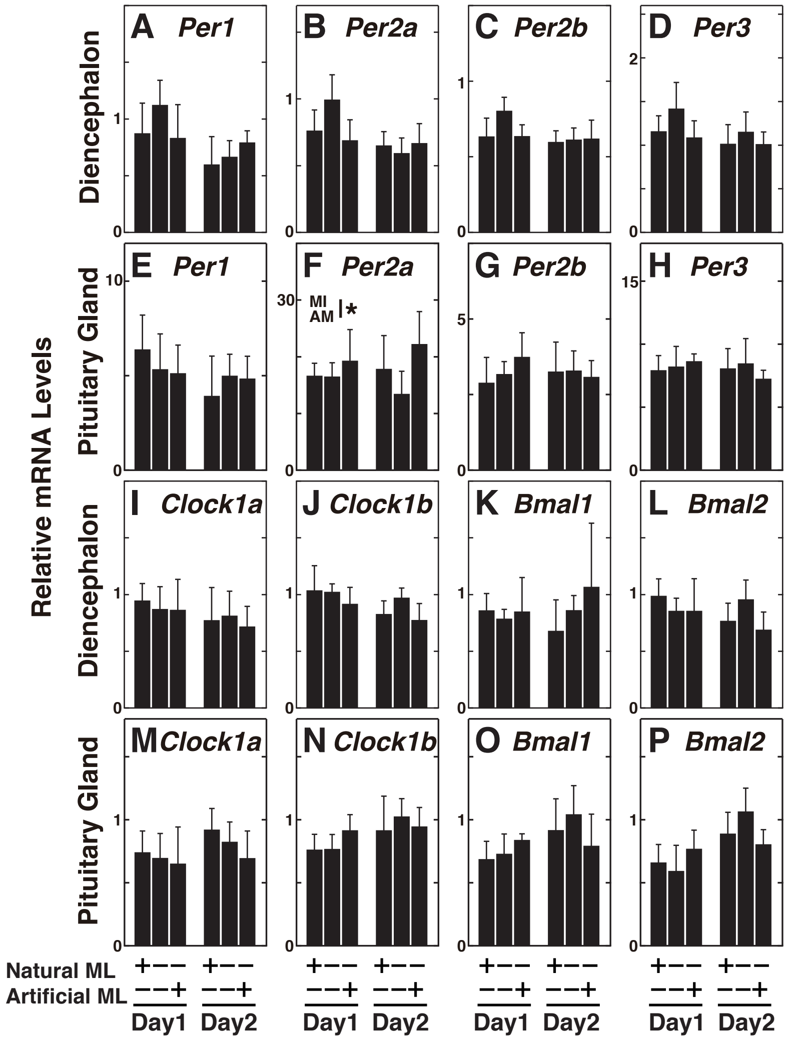

Figure S5. Acute effects of interrupted moonlight on *Per*, *Clock*, and *Bmal* gene expressions in the diencephalon and the pituitary gland. Sampling schedule and nocturnal light conditions were shown in Fig. 7A. The relative expression levels of *Per*, *Clock*, and *Bmal* genes in the diencephalon (A-D, I-L) and pituitary gland (E-H, M-P) were measured under three different nocturnal conditions. Presence or absence of natural moonlight and artificial moonlight is indicated by + or – along the x-axis. Asterisk indicates a significant difference among nocturnal light conditions (two-way factorial ANOVA followed by Tukey's HSD test, $p < 0.05$

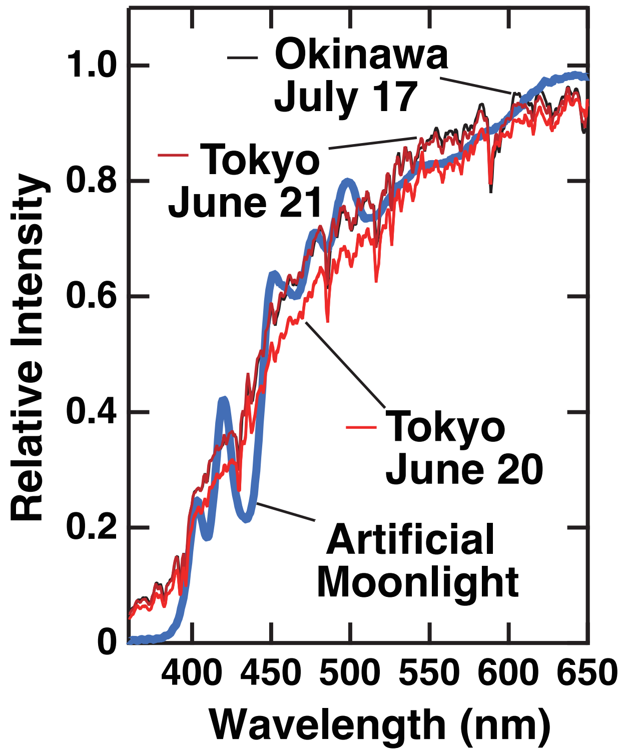

Figure S6. Spectra of natural and artificial moonlight.
The natural moonlight spectrum was measured using a photonic multichannel spectral analyzer (Hamamatsu Photonics, Model PMA-11; type C7473-36) in Tokyo (Suginami city) on June 20 and 21, 2016 (light and dark red lines; Lunar phases 14.5 and 15.5, respectively). An apparatus to measure artificial moonlight was constructed using five power LEDs (UV power LED OSV5XME1C1E (403nm) Optosupply; 3W-UV-Ultra-Violet-420nm, LED generic; Royal Blue LED LuxeonK2; Cyan LuxeonK2; 1W Red-Violet Power LED OSR7XNE1E1E, Optosupply) and five 5mm LEDs (Warm White OSM54K5111A; Cyan OSC34L5111A; Blue OSUB5161A; Lime Green OSC64L5111A; Lavender OSCD4L5111A, all supplied by Optosupply). The combinatorial LED emissions (blue line) were tuned to fit the moonlight spectrum in Tokyo. Moonlight spectrum was also measured at the Sesoko station in Okinawa during an experiment on July 17, 2016 (black line; Lunar phase 12.2). The spectrum was similar to the spectrum in Tokyo on June 21, 2016, and both profiles were roughly reproduced using artificial moonlight.

Table S1 Summary of statistical analysis by three-way ANOVA for evaluating the effect of lunar phase, nocturnal light condition, and Zeitgeber time on the clock gene expressions in the diencephalon of the goldlined spinefoot.

Gene	Lunar Phase (LP)	LPvsZTvsNLC	LP vs ZT (Moonlignt Exposed)			LP vs ZT (Moonlight Interrupted)			NLC vs ZT (Moon Phase fixed)						
		three-way interaction	simple interaction	LP effect	ZT effect	simple interaction	LP effect	ZT effect	simple interaction	NLC effect	ZT effect	ME vs MI (Student t-test)			
												ZT0	ZT6	ZT12	ZT18
Cry1a	NM	F(9, 112)=1.046, p=0.408864	F(9, 64)=1.051, p=0.41095	F(3, 64)=5.097, p=0.00317	F(3, 64)=49.634, p<2e-16	F(9, 48)=0.968, p=0.4776	F(3, 48)=2.510, p=0.0698	F(3, 48)=21.759, p=4.86e-09	F(3, 28)=0.560, p=0.646	F(1, 28)=0.633, p=0.433	F(3, 28)=17.094, p=1.67e-06	no significant interaction			
	FQM								F(3, 28)=0.600, p=0.62022	F(1, 28)=8.677, p=0.00642	F(3, 28)=20.852, p=2.67e-07	no significant interaction			
	FM								F(3, 28)=2.892, p=0.05294	F(1, 28)=10.081, p=0.00363	F(3, 28)=19.075, p=6.16e-07	no significant interaction			
	LQM								F(3, 28)=2.913, p=0.0518	F(1, 28)=1.972, p=0.1713	F(3, 28)=14.951, p=5.32e-06	no significant interaction			
Cry1b	NM	F(9, 112)=1.646, p=0.110732	F(9, 64)=1.495, p=0.1691	F(3, 64)=2.401, p=0.0758	F(3, 64)=73.779, p<2e-16	F(9, 48)=2.356, p=0.0271	F(3, 48)=1.188, p=0.3242	F(3, 48)=43.871, p=8.52e-14	F(3, 28)=0.604, p=0.6181	F(1, 28)=4.304, p=0.0473	F(3, 28)=29.403, p=8.48e-09	no significant interaction			
	FQM								F(3, 28)=2.506, p=0.0794	F(1, 28)=0.061, p=0.8065	F(3, 28)=27.673, p=1.6e-08	no significant interaction			
	FM								F(3, 28)=6.510, p=0.00176	F(1, 28)=0.075, p=0.78647	F(3, 28)=47.919, p=3.74e-11	W=15, p=0.2857	t(7)=1.9558, p=0.09138	t(7)=-0.049836, p=0.9616	t(7)=-10.001, p=2.138e-05
	LQM								F(3, 28)=3.610, p=0.0254	F(1, 28)=1.479, p=0.234	F(3, 28)=19.319, p=5.48e-07	t(7)=-0.74226, p=0.4821	t(7)=1.8161, p=0.1122	t(7)=-0.28185, p=0.7862	W=1, p=0.03175
Cry2	NM	F(9, 112)=1.879, p=0.06221	F(9, 64)=0.346, p=0.95572	F(3, 64)=5.639, p=0.00171	F(3, 64)=31.557, p=1.21e-12	F(9, 48)=1.912, p=0.0727	F(3, 48)=9.616, p=4.42e-05	F(3, 48)=20.632, p=9.94e-09	F(3, 28)=3.199, p=0.03854	F(1, 28)=12.673, p=0.00135	F(3, 28)=7.731, p=0.00065	t(7)= -2.9812, p=0.02048	W=8, p=0.7302	t(7)=-2.8156, p=0.02594	t(7)=-0.031426, p=0.9758
	FQM								F(3, 28)=0.979, p=0.417	F(1, 28)=1.106, p=0.302	F(3, 28)=15.189, p=4.66e-06	no significant interaction			
	FM								F(3, 28)=0.22, p=0.882	F(1, 28)=2.04, p=0.164	F(3, 28)=15.91, p=3.13e-06	no significant interaction			
	LQM								F(3, 28)=2.604, p=0.0716	F(1, 28)=1.922, p=0.1766	F(3, 28)=36.436, p=8.38e-10	no significant interaction			
Cry3	NM	F(9, 112)=0.713, p=0.6964	F(9, 64)=0.864, p=0.56187	F(3, 64)=11.380, p=4.48e-06	F(3, 64)=5.649, p=0.00169	F(9, 48)=1.778, p=0.09723	F(3, 48)=5.262, p=0.00321	F(3, 48)=1.463, p=0.23632	F(3, 28)=0.136, p=0.9379	F(1, 28)=57.488, p=2.93e-08	F(3, 28)=3.693, p=0.0234	no significant interaction			
	FQM								F(3, 28)=1.794, p=0.171	F(1, 28)=20.732, p=9.4e-05	F(3, 28)=1.631, p=0.204	no significant interaction			
	FM								F(3, 28)=0.445, p=0.723	F(1, 28)=2.456, p=0.128	F(3, 28)=1.224, p=0.319	no significant interaction			
	LQM								F(3, 28)=1.396, p=0.2645	F(1, 28)=9.527, p=0.00453	F(3, 28)=6.014, p=0.00269	no significant interaction			
Per1	NM	F(9, 112)=0.854, p=0.5681	F(9, 64)=1.659, p=0.118	F(3, 64)=2.029, p=0.119	F(3, 64)=106.06, p<2e-16	F(9, 48)=1.776, p=0.09782	F(3, 48)=4.501, p=0.00732	F(3, 48)=53.321, p=2.59e-15	F(3, 28)=3.515, p=0.02797	F(1, 28)=9.826, p=0.00401	F(3, 28)=44.431, p=8.59e-11	t(7)=2.2429, p=0.05982	t(7)=2.8408, p=0.02502	t(7)=-1.3896, p=0.2072	t(7)=1.3338, p=0.224
	FQM								F(3, 28)=5.016, p=0.00658	F(1, 28)=1.342, p=0.25638	F(3, 28)=24.708, p=5.07e-08	t(7)=1.9722, p=0.0892	t(7)=-0.27518, p=0.7911	t(7)=-3.6041, p=0.008692	t(7)=-2.1791, p=0.06573
	FM								F(3, 28)=8.647, p=0.000321	F(1, 28)=7.41, p=0.011032	F(3, 28)=39.438, p=3.47e-10	t(7)=2.4826, p=0.04205	t(7)=5.3843, p=0.001026	W=10, p=1	t(7)=-3.7572, p=0.007101
	LQM								F(3, 28)=6.316, p=0.00208	F(1, 28)=0.017, p=0.898	F(3, 28)=40.892, p=2.31e-10	t(7)=1.4808, p=0.1822	t(7)=2.82, p=0.02577	t(7)=-2.8963, p=0.02311	W=0, p=0.01587
Per2a	NM	F(9, 112)=0.864, p=0.5598	F(9, 64)=0.137, p=0.998	F(3, 64)=2.134, p=0.105	F(3, 64)=37.845, p=3.54e-14	F(9, 48)=1.664, p=0.12435	F(3, 48)=2.381, p=0.08115	F(3, 48)=6.100, p=0.00133	F(3, 28)=0.317, p=0.812881	F(1, 28)=4.916, p=0.034904	F(3, 28)=8.847, p=0.000276	no significant interaction			
	FQM								F(3, 28)=8.448, p=0.000373	F(1, 28)=0.115, p=0.736624	F(3, 28)=12.654, p=2.08e-05	t(7)=2.4358, p=0.04503	t(7)=0.44412, p=0.6704	t(7)=-4.1235, p=0.00444	t(7)=-0.46995, p=0.6527
	FM								F(3, 28)=3.879, p=0.01946	F(1, 28)=9.674, p=0.00427	F(3, 28)=11.145, p=5.48e-05	t(7)=2.9992, p=0.01996	t(7)=2.4313, p=0.04533	t(7)=-0.19197, p=0.8532	t(7)=-0.1728, p=0.8677
	LQM								F(3, 28)=2.593, p=0.0724	F(1, 28)=0.035, p=0.8521	F(3, 28)=11.200, p=5.28e-05	no significant interaction			
Per2b	NM	F(9, 112)=0.842, p=0.578961	F(9, 64)=1.005, p=0.446	F(3, 64)=0.597, p=0.619	F(3, 64)=47.026, p=3.53e-16	F(9, 48)=2.266, p=0.033205	F(3, 48)=21.614, p=5.32e-09	F(3, 48)=8.563, p=0.000117	F(3, 28)=0.362, p=0.78101	F(1, 28)=6.012, p=0.020709	F(3, 28)=9.289, p=0.000199	no significant interaction			
	FQM								F(3, 28)=1.600, p=0.2117	F(1, 28)=5.106, p=0.0318	F(3, 28)=23.471, p=8.46e-08	no significant interaction			
	FM								F(3, 28)=0.772, p=0.519197	F(1, 28)=0.007, p=0.935763	F(3, 28)=10.096, p=0.000112	no significant interaction			
	LQM								F(3, 28)=0.58, p=0.63325	F(1, 28)=12.34, p=0.00152	F(3, 28)=45.28, p=7.2e-11	no significant interaction			
Per3	NM	F(9, 112)=0.074, p=0.530186	F(9, 64)=0.836, p=0.586	F(3, 64)=2.014, p=0.121	F(3, 64)=114.584, p<2e-16	F(9, 48)=1.828, p=0.0872	F(3, 48)=2.208, p=0.0992	F(3, 48)=75.609, p<2e-16	F(3, 28)=1.494, p=0.238	F(1, 28)=2.587, p=0.119	F(3, 28)=52.237, p=1.36e-11	no significant interaction			
	FQM								F(3, 28)=0.362, p=0.78122	F(1, 287)=16.681, p=0.000335	F(3, 28)=35.350, p=1.17e-09	no significant interaction			
	FM								F(3, 28)=2.216, p=0.1083	F(1, 28)=10.451, p=0.00313	F(3, 28)=35.384, p=1.16e-09	no significant interaction			
	LQM								F(3, 28)=4.666, p=0.00912	F(1, 28)=24.721, p=2.99e-05	F(3, 28)=66.225, p=7.85e-13	t(7)=-4.0745, p=0.004723	t(7)=-0.37949, p=0.7156	t(7)=-3.0149, p=0.01953	W=0, p=0.01587
Clock1a	NM	F(9, 112)=1.167, p=0.3231	F(9, 64)=0.514, p=0.859	F(3, 64)=0.812, p=0.492	F(3, 64)=171.201, p<2e-16	F(9, 48)=0.88, p=0.5495	F(3, 48)=2.516, p=0.0693	F(3, 48)=36.286, p=2.14e-12	F(3, 28)=7.836, p=0.000598	F(1, 28)=48.364, p=1.46e-07	F(3, 28)=24.167, p=6.33e-08	W=0, p=0.01587	W=0, p=0.01587	W=0, p=0.01587	t(7)=-2.0242, p=0.08262
	FQM								F(3, 28)=3.296, p=0.03492	F(1, 28)=12.259, p=0.00157	F(3, 28)=13.692, p=1.1e-05	W=1, p=0.03175	W=0, p=0.01587	W=1, p=0.03175	t(7)=-0.61614, p=0.5573
	FM								F(3, 28)=2.442, p=0.0851	F(1, 28)=20.984, p=8.72e-05	F(3, 28)=19.085, p=6.13e-07	no significant interaction			
	LQM								F(3, 28)=6.475, p=0.00181	F(1, 28)=78.900, p=1.23e-09	F(3, 28)=80.026, p=7.57e-14	t(7)=-4.5074, p=0.002773	W=0, p=0.01587	t(7)=-6.3209, p=0.0003961	t(7)=-5.7558, p=0.0006943
Clock1b	NM	F(9, 112)=2.331, p=0.019061	F(9, 64)=0.634, p=0.7640	F(3, 64)=2.191, p=0.0976	F(3, 64)=19.754, p=3.54e-09	F(9, 48)=2.863, p=0.00872	F(3, 48)=10.762, p=1.59e-05	F(3, 48)=17.479, p=8.32e-08	F(3, 28)=1.228, p=0.31792	F(1, 28)=9.865, p=0.00395	F(3, 28)=2.020, p=0.13393	no significant interaction			
	FQM								F(3, 28)=4.427, p=0.0114	F(1, 28)=2.880, p=0.1008	F(3, 28)=16.979, p=1.77e-06	W=13, p=0.5556	t(7)=-2.6704, p=0.03198	W=2, p=0.06349	t(7)= 2.6301, p=0.03391
	FM								F(3, 28)=0.166, p=0.918672	F(1, 28)=0.229, p=0.635756	F(3, 28)=8.813, p=0.000283	no significant interaction			
	LQM								F(3, 28)=2.470, p=0.0825	F(1, 28)=7.211, p=0.0120	F(3, 28)=15.835, p=3.26e-06	no significant interaction			
Bmal1	NM	F(9, 112)=1.242, p=0.27709	F(9, 64)=0.337, p=0.959	F(3, 64)=1.878, p=0.142	F(3, 64)=188.623, p<2e-16	F(9, 48)=1.598, p=0.1429	F(3, 48)=2.799, p=0.0499	F(3, 48)=54.705, p=1.61e-15	F(3, 28)=3.560, p=0.0267	F(1, 28)=2.809, p=0.1049	F(3, 28)=34.243, p=1.66e-09	t(7)=-2.1692, p=0.0667	t(7)=-0.53115, p=0.6117	t(7)=2.044, p=0.08023	t(7)=1.4724, p=0.1844
	FQM								F(3, 28)=1.158, p=0.343	F(1, 28)=0.025, p=0.874	F(3, 28)=33.523, p=2.09e-09	no significant interaction			
	FM								F(3, 28)=2.084, p=0.125	F(1, 28)=1.617, p=0.214	F(3, 28)=73.898, p=2.04e-13	no significant interaction			
	LQM								F(3, 28)=0.942, p=0.433	F(1, 28)=0.114, p=0.738	F(3, 28)=91.761, p=1.35e-14	no significant interaction			
Bmal2	NM	F(9, 112)=0.389, p=0.93804	F(9, 64)=0.926, p=0.508658	F(3, 64)=7.885, p=0.000148	F(3, 64)=156.656, p<2e-16	F(9, 48)=0.446, p=0.902	F(3, 48)=0.428, p=0.734	F(3, 48)=34.596, p=4.69e-12	F(3, 28)=2.276, p=0.102	F(1, 28)=0.061, p=0.806	F(3, 28)=32.350, p=3.07e-09	no significant interaction			
	FQM								F(3, 28)=1.950, p=0.145	F(1, 28)=0.241, p=0.627	F(3, 28)=22.122, p=1.51e-07	no significant interaction			
	FM								F(3, 28)=2.055, p=0.12885	F(1, 28)=9.377, p=0.00481	F(3, 28)=31.960, p=3.5e-09	no significant interaction			
	LQM								F(3, 28)=0.869, p=0.469	F(1, 28)=1.814, p=0.189	F(3, 28)=49.570, p=2.52e-11	no significant interaction			

Table S2 Summary of statistical analysis by three-way ANOVA for evaluating the effect of lunar phase, nocturnal light condition, and Zeitgeber time on the clock gene expressions in the pituitary gland of the goldlined spinefoot.

Gene	Lunar Phase (LP)	LPvsZTvsNLC	LP vs ZT (Moonlignt Exposed)			LP vs ZT (Moonlight Interrupted)			NLC vs ZT (Moon Phase fixed)						
												ME vs MI (Student t-test)			
		three-way interaction	simple interaction	LP effect	ZT effect	simple interaction	LP effect	ZT effect	simple interaction	NLC effect	ZT effect	ZT0	ZT6	ZT12	ZT18
Cry1a	NM	F(9, 110)=1.400, p=0.1967	F(9, 62)=2.271, p=0.02855	F(3, 62)=4.972, p=0.00372	F(3, 62)=88.791, p<2e-16	F(9, 48)=1.317, p=0.253	F(3, 48)=1.197, p=0.321	F(3, 48)=86.114, p<2e-16	F(3, 27)=4.072, p=0.0165	F(1, 27)=56.411, p=4.44e-08	F(3, 27)=49.778, p=3.93e-11	t(7)=-2.4841, p=0.04196	t(7)=-5.225, p=0.001219	t(7)=-6.3893, p=0.000371	t(6)=-3.7387, p=0.009638
	FQM								F(3, 28)=2.137, p=0.118	F(1, 28)=102.058, p=7.68e-11	F(3, 28)=45.337, p=7.10e-11	no significant interaction			
	FM								F(3, 28)= 8.16, p=0.000465	F(1, 28)=156.87, p=5.39e-13	F(3, 28)=47.52, p=4.12e-11	t(7)=-5.1224, p=0.001365	t(7)=-7.7855, p=0.0001084	t(7)=-8.6759, p=5.412e-05	t(7)=-5.3965, p=0.001012
	LQM								F(3, 27)=0.771, p=0.52	F(1, 27)=70.284, p=5.40e-09	F(3, 27)=35.961, p=1.42e-09	no significant interaction			
Cry1b	NM	F(9, 110)=1.011, p=0.43573	F(9, 62)=1.823, p=0.0818	F(3, 63)=1.539, p=0.2134	F(3, 62)=76.680, p<2e-16	F(9, 48)=2.307, p=0.0303	F(3, 48)=2.428, p=0.0768	F(3, 48)=100.361, p<2e-16	F(3, 27)=0.328, p=0.805	F(1, 27)=2.150, p=0.154	F(3, 27)=30.254, p=8.75e-09	no significant interaction			
	FQM								F(3, 28)=2.219, p=0.108	F(1, 28)=7.237, p=0.0119	F(3, 28)=55.887, p=6.09e-12	no significant interaction			
	FM								F(3, 28)=3.116, p=0.041965	F(1, 28)=18.029, p=0.000217	F(3, 28)=121.526, p=3.69e-16	t(7)=-4.3703, p=0.003273	t(7)=-0.28118, p=0.7867	t(7)=-2.1024, p=0.07361	t(7)=-8.378, p=6.78e-05
	LQM								F(3, 27)=0.735, p=0.5403	F(1, 27)=4.045, p=0.0544	F(3, 27)=44.150, p=1.51e-10	no significant interaction			
Cry2	NM	F(9, 110)=0.866, p=0.557735	F(9, 62)=0.791, p=0.625	F(3, 63)=1.081, p=0.364	F(3, 62)=42.128, p=5.72e-15	F(9, 48)=1.120, p=0.367564	F(3, 48)=6.794, p=0.000656	F(3, 48)=80.670, p<2e-16	F(3, 27)=2.42, p=0.0879	F(1, 27)=32.46, p=4.71e-06	F(3, 27)=40.16, p=4.31e-10	no significant interaction			
	FQM								F(3, 28)=0.503, p=0.683	F(1, 28)=1.376, p=0.251	F(3, 28)=29.350, p=8.64e-09	no significant interaction			
	FM								F(3, 28)=5.191, p=0.00561	F(1, 28)=29.099, p=9.45e-06	F(3, 28)=24.319, p=5.95e-08	W=3, p=0.1111	t(7)=-2.9315, p=0.02198	t(7)=-3.6249, p=0.008455	t(7)=-2.4648, p=0.04316
	LQM								F(3, 27)=2.319, p=0.0979	F(1, 27)=45.254, p=3.19e-07	F(3, 27)=35.110, p=1.84e-09	no significant interaction			
Cry3	NM	F(9, 110)=1.058, p=0.400	F(9, 62)=1.013, p=0.4397	F(3, 62)=2.287, p=0.0873	F(3, 62)=14.468, p=2.99e-07	F(9, 48)=1.284, p=0.27	F(3, 48)=0.560, p=0.644	F(3, 48)=22.640, p=2.81e-09	F(3, 27)=0.585, p=0.62995	F(1, 27)=6.001, p=0.021067	F(3, 27)=9.884, p=0.000143	no significant interaction			
	FQM								F(3, 28)=1.267, p=0.304749	F(1, 28)=1.385, p=0.249086	F(3, 28)=9.252, p=0.000205	no significant interaction			
	FM								F(3, 28)=0.959, p=0.42593	F(1, 28)=12.089, p=0.00167	F(3, 28)=4.270, p=0.0133	no significant interaction			
	LQM								F(3, 27)=2.047, p=0.130888	F(1, 27)=20.700, p=0.000102	F(3, 27)=15.737, p=4.09e-06	no significant interaction			
Per1	NM	F(9, 110)=1.328, p=0.2305	F(9, 62)=1.751, p=0.0963	F(3, 62)=2.790, p=0.0478	F(3, 62)=66.667, p<2e-16	F(9, 48)=1.160, p=0.342	F(3, 48)=0.458, p=0.713	F(3, 48)=68.435, p<2e-16	F(3, 27)=0.651, p=0.589	F(1, 27)=0.751, p=0.394	F(3, 27)=20.018, p=4.92e-07	no significant interaction			
	FQM								F(3, 28)=0.695, p=0.5627	F(1, 28)=5.919, p=0.0216	F(3, 28)=50.862 p=1.86e-11	no significant interaction			
	FM								F(3, 28)=3.403, p=0.031296	F(1, 28)=17.700, p=0.000241	F(3, 28)=39.276, p=3.63e-10	t(7)=-3.0784, p=0.01786	t(7)=-0.29256, p=0.7783	t(7)=-1.2418, p=0.2543	t(7)=-5.9134, p=0.0005915
	LQM								F(3, 27)=1.347, p=0.28013	F(1, 27)=9.436, p=0.00481	F(3, 27)=43.367, p=1.85e-10	no significant interaction			
Per2a	NM	F(9, 110)=1.346, p=0.2219	F(9, 62)=1.326, p=0.2421	F(3, 62)=0.818, p=0.48883	F(3, 62)=5.691, p=0.00165	F(9, 48)=1.827, p=0.08747	F(3, 48)=3.184, p=0.03205	F(3, 48)=5.064, p=0.00397	F(3, 27)=1.661, p=0.19892	F(1, 27)=9.689, p=0.00435	F(3, 27)=3.697, p=0.02379	no significant interaction			
	FQM								F(3, 28)=0.096, p=0.9614	F(1, 28)=4.198, p=0.0499	F(3, 28)=1.741, p=0.1813	no significant interaction			
	FM								F(3, 28)=1.773, p=0.17511	F(1, 28)=59.061, p=3.36e-08	F(3, 28)=5.642, p=0.00374	no significant interaction			
	LQM								F(3, 27)=0.810, p=0.49935	F(1, 27)=21.545, p=7.98e-05	F(3, 27)=6.274, p=0.00227	no significant interaction			
Per2b	NM	F(9, 110)=1.390, p=0.2011	F(9, 62)=1.450, p=0.187	F(3, 62)=0.263, p=0.852	F(3, 62)=68.133, p<2e-16	F(9, 48)=2.240, p=0.0352	F(3, 48)=1.139, p=0.343	F(3, 48)=56.217, p=9.75e-16	F(3, 27)=0.714, p=0.552	F(1, 27)=7.158, p=0.0125	F(3, 27)=16.062, p=3.44e-06	no significant interaction			
	FQM								F(3, 28)=0.615, p=0.611145	F(1, 28)=17.929, p=0.000224	F(3, 28)=34.574, p=1.49e-09	no significant interaction			
	FM								F(3, 28)=6.801, p=0.00138	F(1, 28)=22.096, p=6.29e-05	F(3, 28)=49.911, p=2.32e-11	W=10, p=1	t(7)=-3.8129, p=0.006603	t(7)=-6.2122, p=0.00044	W=0, p=0.01587
	LQM								F(3, 27)=1.987, p=0.139662	F(1, 27)=20.674, p=0.000103	F(3, 27)=54.472, p=1.4e-11	no significant interaction			
Per3	NM	F(9, 110)=1.376, p=0.2077	F(9, 62)=1.973, p=0.0577	F(3, 62)=2.448, p=0.072	F(3, 62)=181.555, p<2e-16	F(9, 48)=1.247, p=0.29	F(3, 48)=1.154, p=0.337	F(3, 48)=125.530, p<2e-16	F(3, 27)=0.557, p=0.648	F(1, 27)=0.000, p=0.996	F(3, 27)=67.598, p=1.12e-12	no significant interaction			
	FQM								F(3, 28)=0.273, p=0.844	F(1, 28)=0.069, p=0.794	F(3, 28)=90.647, p=1.58e-14	no significant interaction			
	FM								F(3, 28)=3.922, p=0.0186	F(1, 28)=0.107, p=0.7456	F(3, 28)=92.870, p=1.16e-14	t(7)=1.5083, p=0.1752	t(7)=1.1783, p=0.2772	t(7)=-3.3803, p=0.01175	t(7)=-6.9448, p=0.0002222
	LQM								F(3, 27)=1.520, p=0.232	F(1, 27)=2.055, p=0.163	F(3, 27)=60.904, p=3.83e-12	no significant interaction			
Clock1a	NM	F(9, 110)=0.700, p=0.7076	F(9, 62)=0.816, p=0.604	F(3, 62)=0.656, p=0.582	F(3, 62)=81.603, p<2e-16	F(9, 48)=2.240, p=0.0352	F(3, 48)=4.639, p=0.00629	F(3, 48)=147.115, p=<2e-16	F(3, 27)=0.463, p=0.711	F(1, 27)=0.036, p=0.851	F(3, 27)=33.483, p=3.04e-09	no significant interaction			
	FQM								F(3, 28)=0.851, p=0.4781	F(1, 28)=3.525, p=0.0709	F(3, 28)=46.837, p=4.87e-11	no significant interaction			
	FM								F(3, 28)=0.294, p=0.83	F(1, 28)=0.080, p=0.78	F(3, 28)=65.869, p=8.38e-13	no significant interaction			
	LQM								F(3, 26)=2.962, p=0.05070	F(1, 26)=9.172, p=0.00549	F(3, 26)=58.219, p=1.14e-11	no significant interaction			
Clock1b	NM	F(9, 110)=0.687, p=0.7191	F(9, 62)=0.972, p=0.47213	F(3, 62)=5.921, p=0.00128	F(3, 62)=56.143, p<2e-16	F(9, 48)=1.281, p=0.27157	F(3, 48)=6.320, p=0.00106	F(3, 48)=119.532, p<2e-16	F(3, 27)=0.345, p=0.792962	F(1, 27)=17.257, p=0.000294	F(3, 27)=26.783, p=3.01e-08	no significant interaction			
	FQM								F(3, 28)=0.227, p=0.877	F(1, 28)=0.511, p=0.481	F(3, 28)=22.595, p=1.23e-07	no significant interaction			
	FM								F(3, 28)=1.916, p=0.14989	F(1, 28)=47.496, p=4.14e-11	F(3, 28)=12.428, p=0.00148	no significant interaction			
	LQM								F(3, 27)=0.460, p=0.7125	F(1, 27)=2.988, p=0.0953	F(3, 27)=71.189, p=6.06e-13	no significant interaction			
Bmal1	NM	F(9, 110)=1.652, p=0.1094	F(9, 62)=2.475, p=0.0175	F(3, 62)=2.002, p=0.1229	F(3, 62)=196.159, p<2e-16	F(9, 48)=1.545, p=0.159	F(3, 48)= 2.143, p=0.107	F(3, 48)=207.184, p=<2e-16	F(3, 27)=1.194, p=0.33076	F(1, 27)=9.656, p=0.00441	F(3, 27)=152.999, p=<2e-16	no significant interaction			
	FQM								F(3, 28)=0.129, p=0.942	F(1, 28)=0.500, p=0.485	F(3, 28)=55.746, p=6.28e-12	no significant interaction			
	FM								F(3, 28)=4.33, p=0.0125	F(1, 28)=26.01, p=2.11e-05	F(3, 28)=96.79, p=6.88e-15	t(7)=-2.4058, p=0.04707	t(7)=-4.0909, p=0.004626	t(7)=-3.0004, p=0.01993	t(7)=-2.2722, p=0.05729
	LQM								F(3, 26)=1.321, p=0.288	F(1, 26)=37.181, p=1.64e-06	F(3, 26)=196.126, p=<2e-16	no significant interaction			
Bmal2	NM	F(9, 109)=1.266, p=0.263455	F(9, 62)=1.032, p=0.42513	F(3, 62)=4.165, p=0.00943	F(3, 62)=150.998, p<2e-16	F(9, 47)=0.971, p=0.4758	F(3, 47)=3.777, p=0.0165	F(3, 47)=141.828, p<2e-16	F(3, 27)=0.620, p=0.6080	F(1, 27)=4.327, p=0.0471	F(3, 27)=53.776, p=1.62e-11	no significant interaction			
	FQM								F(3, 28)=1.412 , p=0.26009	F(1, 28)=8.490, p=0.00695	F(3, 28)=55.636, p=6.43e-12	no significant interaction			
	FM								F(3, 28)=0.049, p=0.985	F(1, 28)=0.001, p=0.981	F(3, 28)=101.831, p=3.6e-15	no significant interaction			
	LQM								F(3, 26)=5.375, p=0.00515	F(1, 26)=11.358, p=0.00236	F(3, 26)=96.841, p=3.12e-14	t(7)=-2.6494, p=0.03297	t(6)=-1.2941, p=0.2432	t(6)=2.9473, p=0.0257	t(7)=2.0476, p=0.07982

Table S3 Cosinor analysis of clock gene expression levels in the diencephalon

Gene	Lunar Phase (LP)	Nocturnal Light Condition (NLC)					
		Moonlight Exposed (ME)			Moonlight Interrupted (MI)		
		Acrophase	SEM	p value	Acrophase	SEM	p value
Cry1a	**NM**	**4.2**	**1.33**	**<0.001**	**4.1**	**1.55**	**0.002**
	**FQM**	**5.4**	**1.33**	**<0.001**	**6.1**	**1.58**	**0.002**
	**FM**	**5.5**	**1.30**	**<0.001**	**4.9**	**1.67**	**0.018**
	**LQM**	**3.9**	**1.34**	**<0.001**	**6.2**	**1.63**	**0.007**
Cry1b	**NM**	**1.4**	**0.99**	**<0.001**	**1.9**	**1.10**	**<0.001**
	**FQM**	**1.5**	**1.06**	**<0.001**	**1.7**	**1.50**	**0.002**
	**FM**	**1.5**	**0.89**	**<0.001**	**23.3**	**1.26**	**<0.001**
	**LQM**	**2.9**	**1.10**	**<0.001**	**23.6**	**1.39**	**0.006**
Cry2	**NM**	**12.7**	**1.38**	**0.006**	**10.9**	**1.30**	**0.031**
	**FQM**	**11.5**	**1.31**	**<0.001**	**9.9**	**1.48**	**0.012**
	**FM**	**11.7**	**1.31**	**<0.001**	**10.8**	**1.44**	**0.003**
	**LQM**	**11.6**	**1.36**	**0.001**	**11.5**	**1.32**	**<0.001**
Cry3	**NM**	**n.d.**	**1.60**	**0.163**	**n.d.**	**1.62**	**0.059**
	**FQM**	**n.d.**	**1.62**	**0.068**	**n.d.**	**1.79**	**0.332**
	**FM**	**n.d.**	**1.64**	**0.440**	**n.d.**	**1.78**	**0.182**
	**LQM**	**23.1**	**1.56**	**0.006**	**n.d.**	**1.80**	**0.059**
Per1	**NM**	**0.1**	**0.99**	**<0.001**	**23.4**	**1.10**	**<0.001**
	**FQM**	**0.2**	**1.06**	**<0.001**	**22.0**	**1.50**	**0.009**
	**FM**	**0.7**	**0.89**	**<0.001**	**21.8**	**1.26**	**<0.001**
	**LQM**	**0.6**	**1.10**	**<0.001**	**22.1**	**1.39**	**<0.001**
Per2a	**NM**	**2.1**	**1.37**	**0.008**	**1.0**	**1.54**	**0.015**
	**FQM**	**1.9**	**1.29**	**<0.001**	**n.d.**	**1.73**	**0.109**
	**FM**	**2.2**	**1.36**	**<0.001**	**n.d.**	**1.80**	**0.413**
	**LQM**	**2.8**	**1.37**	**<0.001**	**n.d.**	**1.77**	**0.145**
Per2b	**NM**	**5.2**	**1.40**	**0.005**	**4.0**	**1.41**	**0.007**
	**FQM**	**6.3**	**1.26**	**<0.001**	**8.1**	**1.39**	**0.001**
	**FM**	**5.4**	**1.38**	**<0.001**	**n.d.**	**1.68**	**0.082**
	**LQM**	**5.9**	**1.27**	**<0.001**	**6.6**	**1.52**	**<0.001**
Per3	**NM**	**2.1**	**0.94**	**<0.001**	**1.3**	**1.08**	**<0.001**
	**FQM**	**2.8**	**1.00**	**<0.001**	**2.1**	**1.33**	**<0.001**
	**FM**	**1.9**	**0.87**	**<0.001**	**0.0**	**1.24**	**<0.001**
	**LQM**	**2.6**	**0.87**	**<0.001**	**0.9**	**1.14**	**<0.001**
Clock1a	**NM**	**12.8**	**1.01**	**<0.001**	**11.4**	**1.14**	**0.003**
	**FQM**	**12.3**	**1.08**	**<0.001**	**11.1**	**1.08**	**0.014**
	**FM**	**12.7**	**0.97**	**<0.001**	**11.1**	**1.18**	**0.003**
	**LQM**	**11.9**	**1.06**	**<0.001**	**11.6**	**1.22**	**<0.001**
Clock1b	**NM**	**11.2**	**1.51**	**0.020**	**n.d.**	**1.86**	**0.897**
	**FQM**	**12.8**	**1.44**	**<0.001**	**10.5**	**1.37**	**<0.001**
	**FM**	**12.3**	**1.44**	**0.003**	**12.7**	**1.57**	**0.013**
	**LQM**	**12.9**	**1.50**	**0.015**	**12.5**	**1.57**	**<0.001**
Bmal1	**NM**	**11.8**	**0.81**	**<0.001**	**10.4**	**1.06**	**0.001**
	**FQM**	**12.2**	**0.92**	**<0.001**	**11.1**	**1.48**	**0.001**
	**FM**	**12.2**	**0.80**	**<0.001**	**11.1**	**1.06**	**<0.001**
	**LQM**	**10.7**	**0.70**	**<0.001**	**11.3**	**1.17**	**<0.001**
Bmal2	**NM**	**13.2**	**1.19**	**<0.001**	**11.4**	**1.54**	**0.006**
	**FQM**	**13.1**	**1.20**	**<0.001**	**11.1**	**1.25**	**0.009**
	**FM**	**11.0**	**1.16**	**0.001**	**11.0**	**1.35**	**0.001**
	**LQM**	**12.3**	**1.13**	**<0.001**	**11.6**	**1.29**	**<0.001**

Table S4 Cosinor analysis of clock gene expression levels in the pituitary gland

Gene	Lunar Phase (LP)	Nocturnal Light Condition (NLC)					
		Moonlight Exposed (ME)			Moonlight Interrupted (MI)		
		Acrophase	SEM	p value	Acrophase	SEM	p value
Cry1a	**NM**	**3.1**	**1.22**	**<0.001**	**4.1**	**1.41**	**<0.001**
	**FQM**	**4.4**	**1.34**	**0.001**	**3.6**	**1.55**	**<0.001**
	**FM**	**2.8**	**1.29**	**<0.001**	**4.5**	**1.45**	**<0.001**
	**LQM**	**4.2**	**1.31**	**<0.001**	**5.1**	**1.51**	**<0.001**
Cry1b	**NM**	**0.5**	**0.59**	**<0.001**	**1.1**	**0.91**	**<0.001**
	**FQM**	**0.7**	**0.75**	**<0.001**	**0.6**	**0.86**	**<0.001**
	**FM**	**1.7**	**0.69**	**<0.001**	**0.7**	**0.96**	**<0.001**
	**LQM**	**1.5**	**0.73**	**<0.001**	**1.2**	**1.15**	**<0.001**
Cry2	**NM**	**11.0**	**1.35**	**<0.001**	**11.1**	**1.47**	**<0.001**
	**FQM**	**12.4**	**1.37**	**0.001**	**12.4**	**1.45**	**<0.001**
	**FM**	**11.7**	**1.42**	**0.001**	**11.5**	**1.37**	**<0.001**
	**LQM**	**12.4**	**1.32**	**<0.001**	**11.4**	**1.47**	**<0.001**
Cry3	**NM**	**23.5**	**1.49**	**0.005**	**22.8**	**1.74**	**0.003**
	**FQM**	**23.6**	**1.57**	**0.049**	**1.3**	**1.11**	**<0.001**
	**FM**	**n.d.**	**1.57**	**0.110**	**1.5**	**1.18**	**<0.001**
	**LQM**	**22.9**	**1.48**	**0.001**	**22.4**	**1.74**	**0.001**
Per1	**NM**	**24.0**	**1.49**	**<0.001**	**0.5**	**1.74**	**0.005**
	**FQM**	**23.3**	**1.57**	**<0.001**	**23.3**	**1.11**	**<0.001**
	**FM**	**1.0**	**1.57**	**<0.001**	**23.3**	**1.18**	**<0.001**
	**LQM**	**0.9**	**1.48**	**<0.001**	**23.8**	**1.74**	**<0.001**
Per2a	**NM**	**n.d.**	**1.56**	**0.351**	**20.6**	**1.58**	**0.038**
	**FQM**	**n.d.**	**1.57**	**0.208**	**n.d.**	**1.82**	**0.478**
	**FM**	**n.d.**	**1.51**	**0.075**	**n.d.**	**1.78**	**0.160**
	**LQM**	**n.d.**	**1.54**	**0.070**	**n.d.**	**1.75**	**0.228**
Per2b	**NM**	**n.d.**	**1.37**	**0.051**	**5.3**	**1.52**	**0.010**
	**FQM**	**n.d.**	**1.43**	**0.054**	**4.2**	**1.57**	**0.014**
	**FM**	**3.4**	**1.30**	**0.001**	**6.9**	**1.59**	**0.009**
	**LQM**	**5.5**	**1.40**	**<0.001**	**6.5**	**1.54**	**<0.001**
Per3	**NM**	**1.4**	**0.94**	**<0.001**	**1.7**	**1.13**	**<0.001**
	**FQM**	**1.4**	**0.96**	**<0.001**	**1.3**	**1.11**	**<0.001**
	**FM**	**2.2**	**0.78**	**<0.001**	**1.5**	**1.18**	**<0.001**
	**LQM**	**1.4**	**0.96**	**<0.001**	**1.0**	**1.27**	**<0.001**
Clock1a	**NM**	**12.3**	**0.80**	**<0.001**	**11.9**	**1.08**	**<0.001**
	**FQM**	**12.0**	**1.00**	**<0.001**	**12.0**	**1.07**	**<0.001**
	**FM**	**12.2**	**0.84**	**<0.001**	**12.1**	**0.84**	**<0.001**
	**LQM**	**13.4**	**0.85**	**<0.001**	**12.1**	**1.13**	**<0.001**
Clock1b	**NM**	**11.5**	**1.28**	**<0.001**	**12.6**	**1.52**	**<0.001**
	**FQM**	**13.5**	**1.33**	**<0.001**	**13.3**	**1.53**	**<0.001**
	**FM**	**13.5**	**1.30**	**<0.001**	**12.3**	**1.45**	**<0.001**
	**LQM**	**13.2**	**1.28**	**<0.001**	**12.2**	**1.55**	**<0.001**
Bmal1	**NM**	**12.1**	**0.83**	**<0.001**	**12.2**	**1.20**	**<0.001**
	**FQM**	**12.7**	**1.02**	**<0.001**	**12.8**	**1.25**	**<0.001**
	**FM**	**12.8**	**1.02**	**<0.001**	**12.1**	**1.13**	**<0.001**
	**LQM**	**13.2**	**1.02**	**<0.001**	**12.3**	**1.30**	**<0.001**
Bmal2	**NM**	**12.4**	**0.97**	**<0.001**	**12.3**	**1.21**	**<0.001**
	**FQM**	**12.6**	**1.05**	**<0.001**	**12.4**	**1.21**	**<0.001**
	**FM**	**12.2**	**0.99**	**<0.001**	**12.3**	**1.11**	**<0.001**
	**LQM**	**13.4**	**0.93**	**<0.001**	**12.3**	**1.44**	**<0.001**

Table S5 Accession numbers of CRYs used in the phylogenetic analysis

Protein name	Accession number
Goldlined spinefood CRY1a	FX985477
Goldlined spinefood CRY1b	BAL72538
Goldlined spinefood CRY2	FX985476
Goldlined spinefood CRY3	BAL72539
Goldlined spinefood CRY6	FX985478
Zebrafish CRY1a	NP_001070765
Zebrafish CRY1b	BAA96847
Zebrafish CRY2a	BAA96848
Zebrafish CRY2b	NP_571867
Zebrafish CRY3	AAH46088
Zebrafish CRY4	BAA96851
Zebrafish CRY6	XP_009291670
Nile tilapia CRY1a	XP_005456675
Nile tilapia CRY1b	XP_005471126
Nile tilapia CRY2a	XP_005477802
Nile tilapia CRY3	XP_013129259
Seabass CRY1	AFP33464
Seabass CRY2	AFP33463
Xenopus tropicalis CRY1	NP_001017311
Xenopus tropicalis CRY2	BAI82612
Xenopus tropicalis CRY4	BAO09600
Xenopus tropicalis CRY6	XP_002938187
Chicken CRY1	AAK61385
Chicken CRY2	AAK61386
Chicken CRY4	NP_001034685
Mouse CRY1	NP_031797
Mouse CRY2	NP_034093
Anopheles gambia CRY1	ABB29886
Danaus plexippus CRY1	AAX58599
Drosophila melanogaster CRY1	AAC83828
Anopheles gambia CRY2	ABB29887
Danaus plexippus CRY2	ABA62409

Table S6 Accession numbers of PERs used in the phylogenetic analysis

Protein name	Accession number
Goldlined spinefood PER1	ABA42096
Goldlined spinefood PER2a	FX985479
Goldlined spinefood PER2b	EF208027
Goldlined spinefood PER3	FX985480
Zebrafish PER1a	NP_001025354
Zebrafish PER1b	NP_997604
Zebrafish PER2	NP_878277
Zebrafish PER3	NP_571659
Nile tilapia PER1b	ENSONIP00000011630
Nile tilapia PER2a	ENSONIP00000012334
Nile tilapia PER2	ENSONIP00000009999
Nile tilapia PER3	ENSONIP00000024320
Seabass PER1	ADI71975
Senegal sole PER2	CAQ86911
Senegal sole PER3	CAQ68365
Xenopus tropicalis PER1	AAI21905
Xenopus tropicalis PER2b	XP_012825562
Xenopus tropicalis PER3	NP_001072696
Mouse PER1	NP_035195
Mouse PER2	NP_035196
Mouse PER3	NP_035197
Drosophila melanogaster PER	NP_525056

Table S7 Accession numbers of CLOCKs used in the phylogenetic analysis

Protein name	Accession number
Gollined spinefoot CLOCK1a	FX985868
Gollined spinefoot CLOCK1b	LC367223
Chicken CLOCK	AAL98708
Mouse CLOCK	NP_031741
Human CLOCK	AAF13733
Xenopus laevis CLOCK	NP_001083854
Xenopus tropicalis CLOCK	NP_001122127
Nile tilapia CLOCK1a	XP_019207337
Nile tilapia CLOCK1b	XP_003452181
Zebrafish CLOCK1a	NP_571032
Zebrafish CLOCK1b	NP_840080
Zebrafish CLOCK2 (NPAS2)	NP_840084
Drosophila melanogaster CLOCK	AAC39101

Table S8 Accession numbers of BMALs used in the phylogenetic analysis

Protein name	Accession number
**Goldlined spinefood BMAL1**	**FX985869**
**Goldlined spinefood BMAL2**	**LC367224**
**Chicken BMAL1**	**NP_001001463**
**Chicken BMAL2**	**NP_989464**
**Human BMAL1a**	**NP_001284653**
**Human BMAL2**	**BAB01485**
**Mouse BMAL1**	**NP_001229977**
**mouse BMAL2**	**ABC79590**
Xenopus laevis BMAL1	**AAW80970**
**Zebrafish BMAL1**	**NP_571652**
**Zebrafish BMAL2**	**NP_571653**
***Drosophila melanogaster* CYCLE**	**NP_524168**
